# Vertical transmission of hepatitis B virus in the WHO African region: a systematic review and meta-analysis

**DOI:** 10.1016/S2214-109X(24)00506-0

**Published:** 2025-02-26

**Authors:** Nicholas Riches, Marc Y R Henrion, Peter MacPherson, Camilla Hahn, Rabson Kachala, Thomas Mitchell, Daniel Murray, Wongani Mzumara, Owen Nkoka, Alison J Price, Jennifer Riches, Aoife Seery, Noel Thom, Anne Loarec, Maud Lemoine, Gibril Ndow, Yusuke Shimakawa, Peyton Thompson, Camille Morgan, Shalini Desai, Philippa Easterbrook, Alexander J Stockdale

**Affiliations:** a Malawi Epidemiology and Intervention Research Unit, Lilongwe, Malawi; b Department of Clinical Sciences, Liverpool School of Tropical Medicine, Liverpool, UK; c Statistical Support Unit, Blantyre, Malawi; d Malawi Liverpool Wellcome Research Programme, Blantyre, Malawi; e School of Health & Wellbeing, University of Glasgow, Glasgow, UK; f Clinical Research Department, London School of Hygiene & Tropical Medicine, London, UK; g Institute of Tropical Medicine, University of Tübingen, Tübingen, Germany; h German Centre for Infection Research, Tübingen, Germany; i Viral Hepatitis Programme, Department of HIV/AIDS, Ministry of Health, Lilongwe, Malawi; j East Sussex Healthcare National Health Service Trust, Bexhill, UK; k Médecins Sans Frontières, Maputo, Mozambique; l Department of Metabolism, Digestion and Reproduction, Imperial College London, London, UK; m Medical Research Council Unit The Gambia, London School of Hygiene & Tropical Medicine, Banjul, The Gambia; n Insitut Pasteur, Université Paris Cité, Unité d'Épidémiologie des Maladies Émergentes, Paris, France; o Department of Pediatrics, Division of Infectious Diseases, University of North Carolina, Chapel Hill, NC, USA; p Global HIV, Hepatitis and STI Programme, WHO, Geneva, Switzerland; q Department of Clinical Infection, Microbiology and Immunology, Institute of Infection, Veterinary and Ecological Sciences, University of Liverpool, Liverpool, UK

## Abstract

**Background:**

More new infections with hepatitis B virus (HBV) occur annually in the WHO African region than in the rest of the world combined. We did a systematic review and meta-analysis to estimate the prevalence of hepatitis B surface antigen (HBsAg) in pregnant women and vertical transmission events in the region.

**Methods:**

In this systematic review and meta-analysis, we searched PubMed, Embase, Scopus, Africa Index Medicus, and Africa Journals Online for publications between Jan 1, 1992, and Jan 7, 2024, with no language restrictions. HBsAg prevalence and vertical transmission (HBsAg positivity in children aged 6–12 months) were estimated with the use of binomial mixed models with logit links, stratified by infant vaccination status. We estimated HBsAg prevalence for subregions of Africa and for the WHO African region by weighting by estimated livebirths for each subregion. We estimated transmission events using WHO and UNICEF vaccine coverage data and UN population estimates.

**Findings:**

We included 113 studies reporting on HBsAg prevalence from 190 983 pregnant women and 11 studies reporting on vertical transmission. HBsAg prevalence in women receiving antenatal care in the WHO African region (based on 2014–23 data) was 6·2% (95% CI 5·3–7·2). No relationship between risk of bias and HBsAg prevalence was observed. In 2022, an estimated 172 000 vertical transmission events (95% CI 82 000–383 000) occurred (0·4% of livebirths), a fall from a peak of 339 000 (149 000–634 000; 1·2% of all livebirths) in 2001. Increasing birth dose vaccination coverage to the WHO target of 90% could reduce vertical transmission by 43·7% (95% CI 11·6–78·0) to 97 000 events per year (95% CI 58 000–160 000). Adding maternal antiviral prophylaxis with 90% coverage could reduce transmission by 86·3% (95% CI 78·4–94·6) to 24 000 events per year (95% CI 14 000–39 000; 0·06% of livebirths) and achieve WHO elimination targets.

**Interpretation:**

Vertical transmission is an important contributor to HBV transmission in the WHO African region. Scaling up of hepatitis B birth dose vaccination and antiviral prophylaxis is urgently needed, which could achieve elimination of vertical transmission.

**Funding:**

Wellcome Trust.

## Introduction

Chronic hepatitis B infection is the leading cause of liver cirrhosis and hepatocellular carcinoma globally.^1^, ^2^ In 2022, hepatitis B virus (HBV) caused an estimated 272 000 deaths and 771 000 new infections in the WHO African region, accounting for 63% of the global total of new infections.^3^ The prevalence of HBV in children younger than 5 years in this region is 2·5% (95% CI 2·1–3·1), which is the highest in the world.^3^

Prevention of vertical transmission of HBV is an important goal, as this type of transmission is a major cause of new infections and is associated with an increased risk of progression to liver disease compared with other transmission routes.^4^ In 2016, the World Health Assembly set an elimination target of hepatitis B surface antigen (HBsAg) prevalence of less than 0·1% in children younger than 5 years by 2030.^3^

Effective and safe interventions are available. Universal HBV vaccination has been recommended by WHO since 1992.^5^ This public health intervention resulted in a major reduction in global HBsAg prevalence among children younger than 5 years, from 4·4% in 1990, to 0·9% in 2020.^6^ The vaccination schedule comprises a key HBV vaccination birth dose (HepB-BD) within 24 h of birth, followed by two to three further doses in infancy.^7^ By 2022, only 35% of African countries had introduced HepB-BD, with coverage of 18% of births, lagging behind the global coverage average of 45%.^8^ Barriers to implementation include a relatively rural population with births frequently occurring outside health-care facilities, logistical difficulties maintaining a cold chain, and until June, 2024, the absence of support for the HepB-BD from Gavi, the Vaccine Alliance.^9^ Vaccine coverage was additionally disrupted by COVID-19.^10^

**Research in context**

**Evidence before this study**

Most chronic hepatitis B infections can be attributed to vertical transmission in the peripartum period or horizontal transmission in early childhood. Universal infant hepatitis B virus (HBV) vaccination has been highly successful in reducing transmission of the virus and new infections in children. Although the 2020 interim global target for less than 1% hepatitis B surface antigen (HBsAg) prevalence in children younger than 5 years was achieved, the prevalence remains at about 2·5% in the WHO African region. An understanding of the prevalence of HBsAg in pregnant women and vertical transmission rates is important to inform priority interventions to meet the HBV elimination target of less than 0·1% among children younger than 5 years by 2030. In addition to searches conducted for this review on Nov 5, 2024, we searched PubMed with synonyms of the terms “hepatitis B”, “pregnant”, “transmission”, “Africa”, and the names of countries in the WHO African region. A list of the search terms is provided in the appendix (pp 3–4). This search identified 31 new studies in addition to the 7749 articles reviewed. We identified four previous systematic reviews, which reviewed HBsAg prevalence data in pregnant women; one review from 2016 also estimated vertical transmission rates from observational studies in Africa. Limitations of these previous reviews include the absence of criteria for representative sampling, no defined age ranges for testing children to ascertain vertical transmission, and no quality standards for HBsAg assays. Evidence is emerging for poor clinical sensitivity of HBsAg rapid diagnostic tests that do not undergo stringent regulatory approval. The 2016 review found no evidence for reduced transmission after hepatitis B birth dose (HepB-BD) relative to the three-dose vaccine (HepB3) starting at 6 weeks. Observational data from rural Cameroon published in 2022 have, in contrast, shown substantial reductions in vertical transmission in infants receiving timely HepB-BD.

**Added value of this study**

We conducted a systematic review and meta-analysis of HBsAg prevalence in adult and adolescent pregnant women and the rate of vertical transmission in the WHO African region, stratified by HepB3 and HepB-BD vaccination status. We required that studies used diagnostic tests with stringent regulatory approval or evaluation studies consistent with WHO prequalification standards, and that studies used representative sampling methods. We included data assessing vertical transmission in infants aged 6–12 months to avoid confounding from horizontal transmission. Previous reviews did not include criteria for the quality of epidemiological representativeness or diagnostic test performance. We required that both were of an acceptable standard. We therefore improved the quality of the estimates through being more selective with our inclusion criteria. Compared with three previous reviews of HBsAg prevalence in pregnant women, we included up to 104 studies in our review that had not been included previously. Compared with the most recent review of vertical transmission, nine additional studies were included in our review. We estimated that among pregnant women attending antenatal care clinics in the WHO African region, HBsAg prevalence was 6·2% (95% CI 5·3–7·2), and we observed a downward change in antenatal HBsAg prevalence over time. The rate of vertical transmission was associated with vaccine status: when excluding cohorts using maternal antiviral prophylaxis, the rate was 3·9% (2·4–6·2) for recipients of a timely HepB-BD (within 24 h), 5·9% (1·9–16·7) for a delayed HepB-BD (between 24 h and 1 week), 7·3% (1·8–25·4) for HepB3 without HepB-BD, and 15·5% (7·5–29·4) for infants who did not receive any HBV vaccination. We evaluated historical trends in vertical transmission, combining our estimates of HBsAg prevalence and transmission rates by vaccine status with UN population estimates and UNICEF vaccination coverage estimates. We estimate that vertical HBV transmission peaked at 339 000 cases (95% CI 149 000–634 000; 1·2% of all livebirths) in 2001, and fell to 172 000 cases (82 000–383 000; 0·4%) in 2022. Increasing HepB-BD coverage to 90% would reduce vertical transmission events to an estimated 97 000 (58 000–160 000) per year—a reduction of 43·7%. Additionally providing maternal antiviral prophylaxis with 90% coverage for women with a high viral load would reduce transmissions to 24 000 (14 000–39 000; 0·06% livebirths)—a reduction of 86·3%.

**Implications of all the available evidence**

Vertical transmission is an important cause of new hepatitis B infections in the WHO African region. Due to insufficient implementation of interventions that prevent vertical HBV transmission, elimination targets are not currently being met. We found that a timely HepB-BD reduces the risk of vertical transmission and that improving HepB-BD coverage to 90% could lead to a relative reduction of transmission events by 44%. Support from Gavi the Vaccine Alliance from July, 2024, for procurement of monovalent birth dose vaccines and programme implementation will help to achieve this goal. Additionally providing maternal antiviral prophylaxis to 90% of eligible women would reduce vertical transmission by 86% and would achieve WHO elimination targets in Africa. HepB-BD alone is insufficient to prevent vertical transmission, and expanded access to maternal antiviral prophylaxis will be required to meet global elimination targets.

Emerging evidence shows that Hep-BD might be insufficient to prevent HBV vertical transmission for women with high HBV DNA (>200 000 IU/mL).^11^ Maternal antiviral prophylaxis (MAP) with tenofovir disoproxil fumarate (TDF) is recommended for pregnant women with high HBV DNA concentrations to reduce the peripartum transmission risk.^12^ Additionally, WHO 2024 guidelines conditionally recommend TDF MAP for all HBsAg-positive pregnant women in settings with little access to HBV DNA quantification.^12^ In randomised trials and observational studies, MAP during pregnancy is a highly effective intervention.^13^ In the last 10 years, additional evidence on the effectiveness of vertical transmission interventions in Africa has become available, though available data remain insufficient.

To generate evidence for action towards the 2030 targets, we reviewed the latest evidence from the WHO African region on HBsAg prevalence in pregnancy and vertical transmission, stratified by HBV vaccination and MAP, and modelled the possible effect of scaling up vaccination to reach the WHO targets.

## Methods

### Search strategy and selection criteria

We performed a systematic review and meta-analysis of the prevalence of HBsAg in pregnant women and adolescent girls (hereafter referred to as pregnant women) resident in the WHO African region. The women were stratified based on the three residence subregions (west, central, and the combined eastern and southern Africa region) and the prevalence of hepatitis B e-antigen (HBeAg). We estimated HBV vertical transmission rates stratified by key interventions: timely HepB-BD (within 24 h), a 3-dose vaccination series not including a birth dose (HepB3), and MAP. We conducted this review according to a protocol (appendix pp 2–7) and PRISMA guidelines.

We searched PubMed, Embase, Scopus, African Index Medicus, and African Journals Online for publications in the 22-year period between Jan 1, 1992, and Jan 7, 2024. A list of the search terms is provided in the appendix (pp 2–7). We excluded studies published before 1992, as they were unlikely to include eligible diagnostic tests. No language restrictions were applied; we obtained translations of publications in languages unknown to the study team. We additionally requested data through the Hepatitis B in Africa Collaborative Network (HEPSANET).

We included surveys that tested pregnant women for HBsAg in the WHO African region with the use of diagnostic tests that fulfilled prespecified quality assurance criteria. These surveys used rapid diagnostic tests (RDTs), which were either WHO prequalified or had stringent regulatory approval from Europe, the USA, Canada, Australia, or Japan; had published validation data fulfilling WHO prequalification criteria; or that used an ELISA or EIA**,** or chemiluminescence immunoassay (CLIA) for HBsAg detection (list of tests in appendix pp 8–11). We applied these criteria in response to evidence of poor sensitivity of HBsAg RDTs not meeting these standards.^14^, ^15^ For studies evaluating HBeAg, we used ELISA or EIA or CLIA data and excluded HBeAg RDT data in view of evidence of poor sensitivity from commercially available RDTs in African populations.^16^, ^17^

We required that surveys described sampling methods and used random sampling or consecutive sampling of eligible women over a specified interval. We included surveys that included people living with HIV if participants were not sampled or stratified based on HIV status. We excluded acute HBV, exclusively people living with HIV, blood donors, or migrant populations. To estimate vertical transmission rates, we included surveys that tested infants aged 6–12 months, to avoid confounding from horizontal transmission among older children.

### Data analysis

Two authors (NR and AS) screened abstracts to identify possibly relevant articles and the full text of articles identified by either investigator. Discordance for inclusion after full-text review was resolved by consensus discussion among NR and AS. We used a bespoke data extraction tool to record population, setting, geography, timing, test type, HBsAg prevalence, and HIV prevalence. Data extraction from HBsAg prevalence and vertical transmission surveys was done independently by two individuals from a team of 11 randomly allocated reviewers chosen from among the authors. Discrepancies between duplicate extractions were resolved independently by a third reviewer (NR, appendix p 6). We assessed the risk of bias by adapting a quality assessment tool checklist with questions relating to sampling methods, testing, and recruitment procedures (appendix p 12).

We modelled HBsAg and HBeAg prevalence for subregions and for HBV vertical transmission using a binomial mixed model with maximum likelihood estimation with adaptive Gauss–Hermite quadrature approximation as implemented in R (meta version 8.0). We estimated HBsAg prevalence in the WHO African region by weighting subregional estimates by the number of livebirths from the UN data for 2022.^18^ For antenatal HBsAg prevalence estimation, we used data published from 2014 to 2024 to consider more recent data, whereas we used the full dataset (1992–2024) for temporal trend analysis and vertical transmission rate modelling. In sensitivity analyses, we compared the estimates from the primary model to a model that incorporated sampling weights for geographical coverage (appendix p 13), weighting country-level estimates as a proportion of the total number of women of reproductive age (15–49 years) in the corresponding WHO subregion (appendix p 14). We assessed for an association between risk of bias and HBsAg prevalence using a principal component analysis to derive a risk of bias score. Multivariable meta-regression was done to investigate associations between HBsAg prevalence and the number of years since national HepB3 introduction, HBsAg test type, and WHO subregion.

To estimate HBV vertical transmission, we stratified estimates with 95% CIs for four groups: timely HepB-BD (administration within 24 h of birth); delayed HepB-BD (between 24 h and 1 week after birth); HepB3 (without a birth dose, commencing after 6 weeks); and unvaccinated. We analysed vertical transmission in studies of women given TDF MAP; data to stratify estimates on both vaccination status and MAP were insufficient.^19^ To facilitate estimation of vertical transmission events, we assumed that infants receiving HepB-BD also completed HepB3. We compared vertical transmission for infants who received HepB-BD, HepB3, and unvaccinated infants using meta-regression with an Empirical Bayes estimator using logit-transformed values. To model temporal trends in HBsAg prevalence, we fitted a mixed-effects meta-regression model with a restricted cubic spline for the mid-study date and WHO region. We used HBsAg prevalence in pregnancy, national vaccine coverage, and livebirths data (appendix p 15) to estimate HBV vertical transmission events with 95% CI between 2000 and 2022. Data on vertical transmission estimates stratified by HBeAg status or HBV DNA thresholds were insufficient; these variables were not included in our transmission model. To estimate vertical transmission events, we multiplied: maternal HBsAg prevalence estimates, the number of births (stratified by infant vaccination status) and vertical transmission estimates for infants receiving HepB-BD, or HepB, or no vaccination, and rounded to the nearest thousand. We excluded data on transmission estimates from cohorts that also used MAP to avoid confounding; there were insufficient data to jointly estimate vertical transmission by vaccine subgroup and MAP. To estimate the potential effect of MAP implementation, we used the pooled effect size from a global meta-analysis of randomised and observational studies.^13^ We derived nested confidence intervals by using parametric bootstrapping with bootComb with 1e6 bootstrap samples in R. Analyses were done with the use of R (version 4.3.1, Vienna, Austria).

### Role of the funding source

The funder of the study had no role in study design, data collection, data analysis, data interpretation, or writing of the report.

## Results

**Figure 1:**
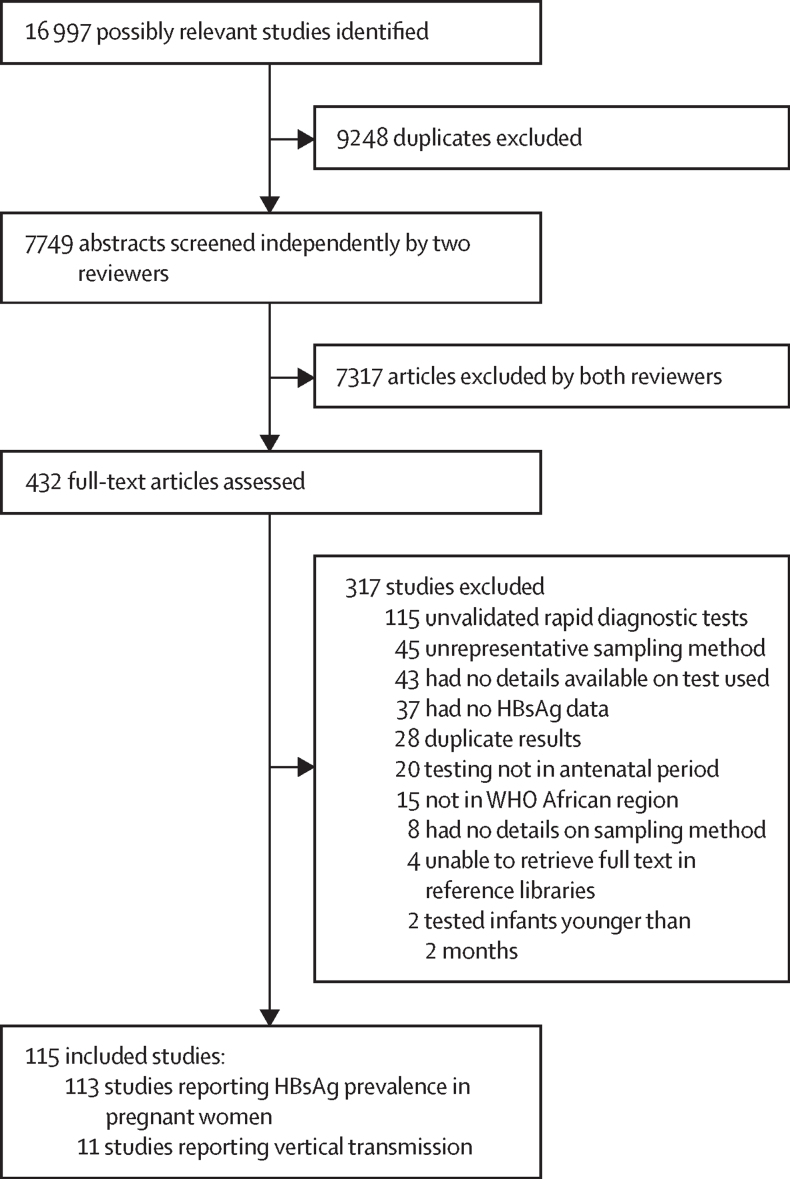
Study selection HBsAg=hepatitis B surface antigen.

Our search retrieved 7749 unique results. After reviewing abstracts, 432 full-text articles were evaluated and 115 articles were included, comprising 104 studies reporting HBsAg prevalence in pregnant women,^20^, ^21^, ^22^, ^23^, ^24^, ^25^, ^26^, ^27^, ^28^, ^29^, ^30^, ^31^, ^32^, ^33^, ^34^, ^35^, ^36^, ^37^, ^38^, ^39^, ^40^, ^41^, ^42^, ^43^, ^44^, ^45^, ^46^, ^47^, ^48^, ^49^, ^50^, ^51^, ^52^, ^53^, ^54^, ^55^, ^56^, ^57^, ^58^, ^59^, ^60^, ^61^, ^62^, ^63^, ^64^, ^65^, ^66^, ^67^, ^68^, ^69^, ^70^, ^71^, ^72^, ^73^, ^74^, ^75^, ^76^, ^77^, ^78^, ^79^, ^80^, ^81^, ^82^, ^83^, ^84^, ^85^, ^86^, ^87^, ^88^, ^89^, ^90^, ^91^, ^92^, ^93^, ^94^, ^95^, ^96^, ^97^, ^98^, ^99^, ^100^, ^101^, ^102^, ^103^, ^104^, ^105^, ^106^, ^107^, ^108^, ^109^, ^110^, ^111^, ^112^, ^113^, ^114^, ^115^, ^116^, ^117^, ^118^, ^119^, ^120^, ^121^, ^122^, ^123^ nine studies reporting HBsAg prevalence and vertical transmission,^124^, ^125^, ^126^, ^127^, ^128^, ^129^, ^130^, ^131^, ^132^ and two studies reporting eligible data on transmission only (figure 1).^133^, ^134^

We contacted 26 study authors to seek clarification for assessing study eligibility and 18 authors provided supplementary data. We excluded 115 studies with unvalidated HBsAg tests. Risk of bias assessment indicated that absence of reporting on the refusal rate among participants (68%), ambiguous eligibility criteria (59%), or failure to describe the study setting and participants in detail (50%) were the most commonly observed issues. Aggregate and study-level assessments are shown in the appendix (pp 16–25).

**Figure 2:**
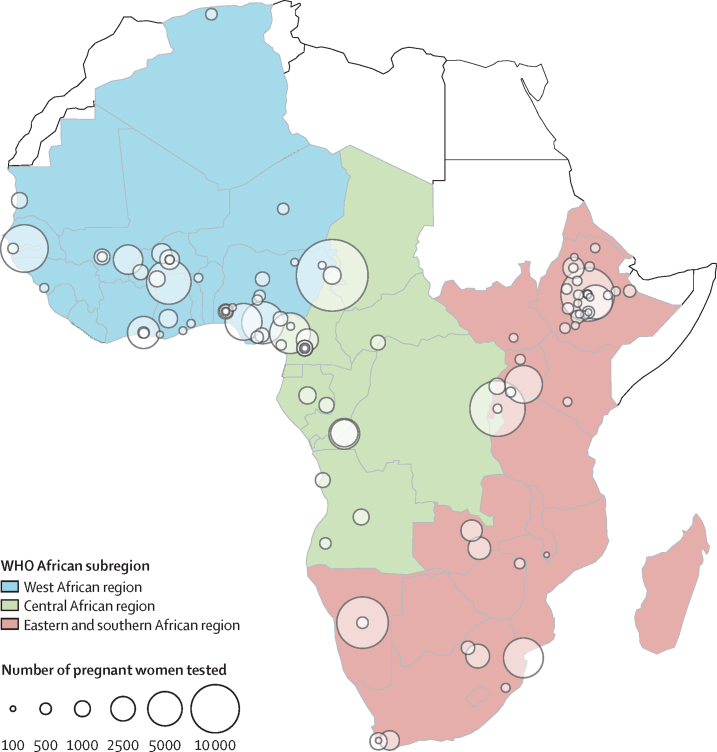
Map of 113 included samples estimating hepatitis B surface antigen prevalence in pregnant women attending for antenatal care

**Figure 3:**
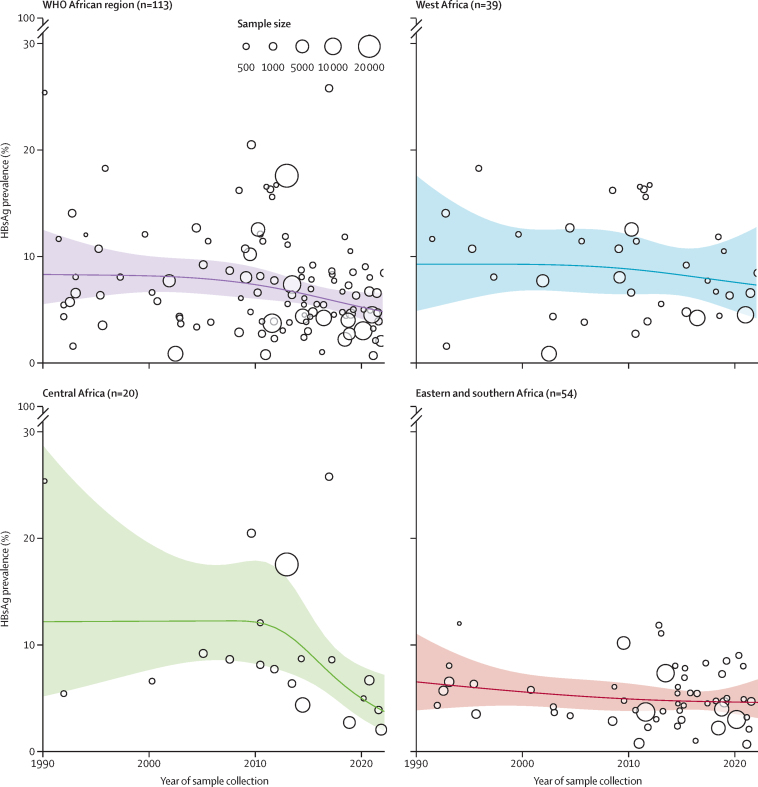
HBsAg prevalence in pregnant women living in the WHO African region and subregions in 1990–2022 Points show HBsAg prevalence estimates of individual studies. Lines show predicted prevalence from a random effects meta-regression model of mid-study date using a restricted cubic spline with three knots. Shaded areas indicate 95% CIs. HBsAg=hepatitis B surface antigen.

The 113 included HBsAg prevalence surveys were from 29 countries in eastern and southern Africa (n=54; 48%), west Africa (n=39, 35%), and central Africa (n=20, 18%; Figure 2, Figure 3). Surveys sampled a median of 498 pregnant women (IQR 299–1186); overall 190 983 women were tested for HBsAg (appendix pp 27–31). The median year of national HepB3 vaccine implementation in included studies was 2004 (range 1994–2014), equating to a median of 8·2 years (IQR 1·6–13·3) since HepB3 introduction. Median (of study means) of participants’ ages was 26·1 (IQR 25·1–27·5). Study median HIV prevalence was 3·5% (IQR 0·6–7·4). HBeAg prevalence data were available in 42 (37·2%) of 113 studies. ELISA and EIA assays were used most commonly for HBsAg diagnosis (78 [69·0%] of 113, rapid diagnostic tests 30 [26·5%] of 113, and CLIAs 5 [4·4%] of 113).

We estimated HBsAg seroprevalence in pregnant women from 72 studies published in 2014–23, including 143 457 participants from 22 countries. Pooled HBsAg seroprevalence was 6·2% (95% CI 5·3–7·2) overall. The seroprevalence was higher in central Africa (7·0% [4·9–10·0%], estimated from 14 surveys) and west Africa (7·5% [5·9–9·4], 17 surveys), than in eastern and southern Africa (4·4% [3·6–5·4], 41 surveys; p=0·016; appendix pp 32–33). Sensitivity analyses including all data (1992–2023) and without the use of sampling weights are shown in the appendix (p 14). No relationship between risk of bias and HBsAg prevalence was observed (appendix p 34).

HBsAg prevalence in pregnant women across the WHO African region declined from 7·3% (95% CI 6·0–8·9) in 2010, to 4·8% (3·5–6·5) in 2022 (figure 3). In a multivariable meta-regression analysis, HBsAg prevalence was not associated with test type (comparing ELISA, CLIA, or RDTs; appendix p 35). A longer time between HBV vaccine introduction and study midpoint was associated with lower maternal HBsAg prevalence (adjusted odds ratio [OR] 0·98; 95% CI 0·97–1·00; p=0·010 per year), adjusting for test type and WHO subregion.

HBeAg in HBsAg-positive pregnant women was assessed in 19 surveys between 2014 and 2023; eight were done in central Africa, seven in eastern and southern Africa, and four in west Africa. HBeAg prevalence was 14·3% (95% CI 10·9–19·5) overall—12·5% (6·6–22·5) for central, 18·1 (11·5–27·2) for west, and 11·4% (6·6–19·1) for eastern and southern Africa (appendix p 36).

Vertical transmission rates were reported in 11 eligible studies from nine countries and included 1280 mother–infant pairs (appendix pp 37–40). Infants were tested for HBsAg at a median age of 8 months (IQR 6–9). Vertical transmission was reported in seven samples including 558 infants receiving timely HepB-BD (within 24 h), four samples including 62 infants who received a delayed birth dose (administered between 24 h and 1 week after delivery), five samples including 250 infants administered HepB3 (without a birth dose), and six samples including 410 infants who were not vaccinated (appendix pp 41–43).

Pooled rates of vertical transmission were 1·8% (95% CI 0·5–6·1) in the timely Hep-BD group, 6·4% (2·4–16·0) for individuals receiving delayed Hep-BD, 6·2% (1·5–21·8) among people who completed HepB3 (without a birth dose), and 11·2% (4·6–25·1) for individuals who were unvaccinated (appendix pp 41–42). Considering cohorts in which vaccination was the sole preventive intervention, after excluding data from three cohorts that also offered MAP, rates of vertical transmission were 3·9% (2·4–6·2) for timely HepB-BD, 5·9% (1·9–16·7) for delayed birth dose, 7·3% (1·8–25·4) for HepB3, and 15·5% (7·5–29·4) for unvaccinated infants (appendix p 43). Infants receiving HepB-BD had a lower rate of vertical transmission than did infants receiving HepB3 without HepB-BD (OR 0·28 [95% CI 0·08–0·92]; p=0·037) or unvaccinated infants (0·19 [0·06–0·55]; p=0·002). Three eligible studies^124^, ^127^, ^131^ reported outcomes for tenofovir MAP for pregnant women with high HBV DNA; among 56 infants, 38 (68%) had received HepB-BD, one (2%) had HepB3 (without HepB-BD), ten (18%) were unvaccinated, and for seven (13%) vaccine status was unknown. A single transmission event occurred among women receiving MAP, resulting in a pooled transmission rate of 1·8% (95% CI 0·3–11·6). This event occurred in the context of a high maternal viral load (8·3 log_10_ IU/mL), remaining high at delivery (7·9 log_10_ IU/mL), and no HepB-BD was administered.^128^

**Figure 4:**
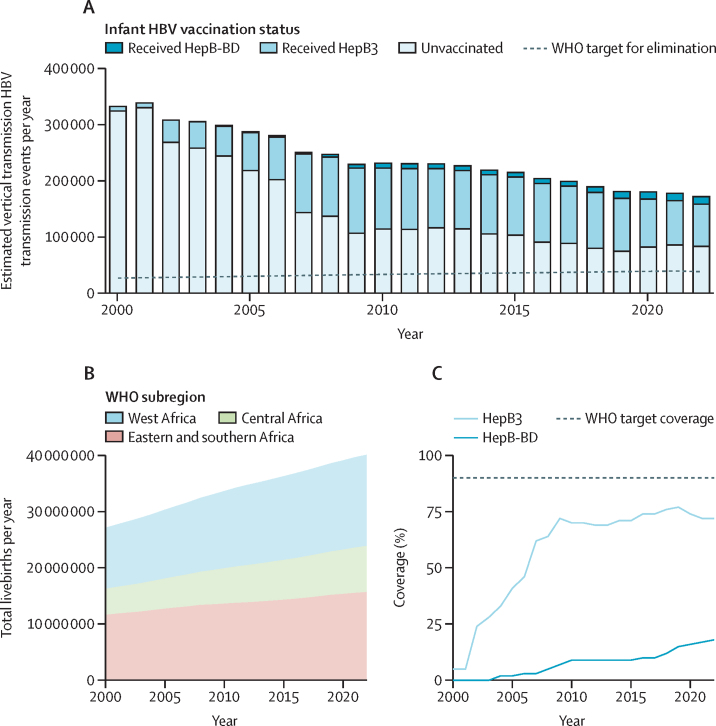
Change in HBV vertical transmission events in the WHO African region in 2000–22 (A) Number of estimated mother-to-child HBV transmission events each year, stratified by infant vaccination status. Dashed lines indicate the WHO elimination targets of hepatitis B surface antigen prevalence of less than 0·1% and HBV vertical transmission of less than 2%. (B) Changes in livebirths over time. Estimates are from UN Population Division statistics.18 (C) Vaccine coverage estimates for HepB-BD and HepB3. Dashed lines show the WHO 90% vaccine coverage target. Vaccine coverage data are from UNICEF and WHO estimates. HBV=hepatitis B virus. HepB-BD=hepatitis B birth dose. HepB3=hepatitis B three-dose vaccine.

Vertical transmission was estimated to have peaked at 339 000 transmission events (95% CI 149 000–634 000; 1·2% of all livebirths) in 2001, and decreased to 172 000 (82 000–383 000; 0·4% of livebirths) in 2022 (figure 4). If HepB3 and Hep-BD coverage in Africa increased to the WHO target of 90%, we estimate that the number of vertical transmission events would fall to 97 000 (58 000–160 000), a reduction of 43·7% representing 0·24% of livebirths. Additionally providing MAP with 90% coverage to women with high viral loads could reduce vertical transmission events by 86·3% to 24 000 (14 000–39 000) per year (0·06% of livebirths), and reach the WHO elimination target (table).

**Table:** Projected vertical transmission events per year for HBV coverage and maternal antiviral prophylaxis scenarios

	**HepB-BD and HepB3**	**HepB3 alone**	**No HBV vaccine**	**Estimated events (95% CI)**	**Transmission rate (total livebirths; 95% CI)**	**Absolute reduction in events, relative to status quo (95% CI)**	**Reduction in events, relative to status quo (95% CI)**
Scenario 1: status quo; coverage for WHO African region in 2022	18%	54%	28%	172 000 (82 000 to 383 000)	0·43% (0·11 to 1·08)	NA	NA
Scenario 2: increasing vaccine coverage to global average for rest of the world	56%	33%	11%	121 000 (65 000 to 250 000)	0·30% (0·10 to 0·71)	52 000 (8000 to 139 000)	30·0% (15·5 to 42·5)
Scenario 3: attaining WHO elimination targets for vaccine coverage	90%	0%	10%	97 000 (58 000 to 160 000)	0·24% (0·14 to 0·40)	75 000 (−9000 to 262 000)	43·7% (11·6 to 78·0)
Scenario 4: attaining WHO elimination coverage targets and use of tenofovir maternal antiviral prophylaxis with 90% coverage	90%	0%	10%	24 000 (14 000 to 39 000)	0·06% (0·04 to 0·10)	149 000 (62 000 to 351 000)	86·3% (78·4 to 94·6)

Infants receiving HepB-BD are assumed to also receive HepB3. The HepB3 category describes infants who have not received a birth-dose vaccine but receive 3 doses commencing from age of 6 weeks. Estimated efficacy of maternal antiviral prophylaxis is derived from a global meta-analysis of randomised and observational studies.^13^ HBV=hepatitis B virus. HepB-BD=hepatitis B birth dose. HepB3=hepatitis B three-dose vaccine. NA=not applicable.

## Discussion

This comprehensive analysis of antenatal HBV prevalence and vertical transmission data from the WHO African region has identified substantial gaps in progress towards HBV elimination targets. Chronic hepatitis B infection remains highly prevalent in women attending for antenatal care in the region, particularly in west and central Africa. Despite improvements in the coverage of HepB3 and HepB-BD, we estimate that 172 000 infants (95% CI 82 000–383 000) were infected through vertical transmission of HBV in 2022, representing 0·4% of livebirths in the WHO African region.^18^ Our findings highlight the need to prioritise rapid scale-up of interventions to reduce vertical transmission. These interventions have the potential to reduce the number of transmissions by 43% each year if HepB-BD and HepB3 reached the 90% vaccine coverage WHO target, and to achieve elimination targets by additionally implementing maternal antiviral prophylaxis with 90% coverage.

We found that vertical transmission falls for timely (<24 h) administration of the HepB-BD vaccine in addition to HepB3, compared with HepB3 alone. We observed that delayed HepB-BD (administration between 24 h and 1 week of life) did not confer reduced transmission relative to HepB3 without HepB-BD. This result reinforces the need for strengthening health-care systems to facilitate timely Hep-BD. Our findings are consistent with an observational study from Cameroon,^11^ in which vertical transmission was 5·6% in children (tested up to the age of 8 years) who had received a HBV vaccine within 24 h of birth, 7% for whom the vaccine was administered between 24 h and 47 h, and 16·7% for individuals who were given the vaccine between 48 h and 96 h. We found that MAP was associated with a very low transmission risk, but available data were scarce. We highlight the need for additional studies focused on the implementation of MAP in Africa, also in settings without access to HBV DNA quantification where 2024 WHO guidelines now conditionally recommend universal MAP from the second trimester of pregnancy, regardless of the viral load of the pregnant woman.^135^

African countries have had persistent challenges in implementing timely Hep-BD. In The Gambia, the first African country to introduce HepB-BD in 1990, birth dose coverage was 26% in 2022,^8^ whereas the population-weighted coverage was 18% across the WHO African region. A complex, diverse, and heterogeneous range of barriers to implementation at the individual, health worker, and health systems levels might drive inequality in access to prevention.^135^ Even when a timely HepB-BD is administered, the risk of HBV vertical transmission has been shown to exceed 30% among women with a high viral load (>200 000 IU/mL); for these women, MAP is required to further reduce transmission risk.^131^ We found that 13·1% (95% CI 11·4–15·0) of pregnant women with HBV were HBeAg positive and would probably require additional MAP. In pilot data from the Democratic Republic of the Congo, MAP for HBV DNA of more than 200 000 IU/mL together with HepB-BD and HepB3 resulted in no transmission events among seven (78%) of nine pregnant women eligible for MAP who had available outcome data (were not lost to follow-up).^131^ Previously poor sensitivity of commercially available HBeAg RDTs in Malawi and west Africa was found.^16^, ^17^ In decentralised rural settings, access to timely viral load testing is often poor. Several factors make wider implementation of MAP feasible in the WHO African region. Experience from HIV prevention programmes, including measures for treatment adherence support, can be leveraged for HBV prevention of vertical transmission, also targeting syphilis as part of a triple elimination strategy.^136^ The emergence of novel tools might assist with MAP implementation including point-of-care hepatitis B core-related antigen RDTs, which in The Gambia were associated with a high sensitivity (91%) for detecting HBV DNA of more than 200 000 IU/mL,^137^ or the use of near-patient HBV DNA quantification with platforms widely available in African countries (GeneXpert, Cepheid, USA).

We observed evidence of a decrease in HBsAg prevalence from 1990 to 2022, and this change is expected to accelerate in coming years as the population of pregnant women increasingly includes individuals born after implementation of the HBV infant vaccination. Declining HBsAg prevalence might also be related to increasing median age at first pregnancy, as older age is associated with decreased HBeAg prevalence and lower vertical transmission risk.^138^

Several limitations of this study relate to the composition and quality of the available data. We have incomplete coverage, with no HBsAg prevalence data for pregnant women available for 20 (42·6%) of 47 countries in the region. Prevalence and transmission estimates were heterogeneous, with evidence of temporal and geographical variation, which is a common finding in meta-analyses of prevalence. Our meta-regression analysis was underpowered to identify associations with prevalence.^139^ Rural areas were under-represented in the available datasets. All included studies reporting vertical transmission were non-randomised observational studies, which lead to a substantial risk of confounding—for example, by an association between vaccination and maternal age or socioeconomic status. There was an overall paucity of data for vertical transmission estimates for vaccine subgroups, which resulted in corresponding uncertainty. We did not have sufficient data to explicitly model transmission stratified by maternal HBeAg status or HBV DNA thresholds. Our estimates for the possible effect of MAP were based on a meta-analysis largely from Asian studies^13^ due to limited data availability from Africa. These data might not reflect real-world performance of MAP in Africa. This limitation highlights the need for implementation studies to observe MAP effectiveness in the WHO African region.

Our study had substantial strengths relative to previous reviews. Of the 113 HBsAg prevalence studies, Bigna and colleagues’ review^140^ did not include 76 (67%) of them, Wondmeneh's review^141^ did not include 88 (78%), and Larebo and colleagues’ review^142^ did not include 104 (92%). For the 11 studies reporting vertical transmission, nine (82%) were new or not previously included in a 2016 review.^143^ Our approach required that included studies used systematic sampling methods, and we restricted to studies that used validated HBsAg diagnostic tests, to minimise selection and information bias. Commercially available rapid diagnostic tests for HBsAg that have not undergone stringent regulatory approval might have a clinical sensitivity as low as 45%,^14^ highlighting the need for assay evaluation. We restricted the assessment of vertical HBV transmission for infants aged 6–12 months, to reduce the possible influence of horizontal transmission, which might be substantial for older and unvaccinated children.^144^ Finally, our binomial mixed model offers advantages compared with previous random effects models in previous reviews, which rely upon data transformations and approximations of a normal distribution.^145^

We believe our findings strengthen the case for urgent, coordinated action to implement HepB-BD at scale across the WHO African region, a population which is currently experiencing the greatest burden of hepatitis B and the least support available to prevent transmission. This action will be facilitated by catalytic funding from Gavi for HepB-BD implementation. Given persisting barriers to implementation of HepB-BD and its incomplete effectiveness for women with high viral loads, our findings highlight the need for a comprehensive vertical transmission strategy to screen all pregnant women for HBsAg and to offer antiviral therapy to those who are most at risk of transmission. This programme should be integrated with strategies to test for and eliminate HIV and syphilis transmission. A clear opportunity exists to transform the outlook for liver disease in the WHO African region and set out on a credible path to elimination of HBV.

### Contributors

### Data sharing

Data collected for this study comprising extracted aggregate data from included studies of hepatitis B surface antigen prevalence and vertical transmission and results from the risk of bias assessment are included in the supplementary appendix. The study protocol is included in the supplementary appendix.

## Declaration of interests

YS has received a research grant and honoraria from Gilead Sciences, and received research materials from Abbott Laboratories and Fujirebio. PT has received donations of diagnostic supplies from Abbott Laboratories. All other authors declare no competing interests.
