# Supplementary material for: Vertical transmission of hepatitis B virus in the WHO African region: a systematic review and meta-analysis
Source: Lancet Glob Health. 2025 Feb 26;13(3):e447–58. doi: 10.1016/S2214-109X(24)00506-0 (PMC11868780; doi:10.1016/S2214-109X(24)00506-0)

THE LANCET
Global Health

Supplementary appendix

This appendix formed part of the original submission and has been peer reviewed. We post it as supplied by the authors.

Supplement to: Riches N, Henrion MYR, MacPherson P, et al. Vertical transmission of hepatitis B virus in the WHO African region: a systematic review and meta-analysis. *Lancet Glob Health* 2025; **13:** e447–58.

Supplementary materials for Vertical transmission of hepatitis B in the World Health Organisation African region: systematic review and meta-analysis

Contents

S1 Appendix: Systematic review protocol 2

S2 Appendix. Details of HBsAg tests used 8

S3 Appendix. Quality assessment checklist 12

S4 Appendix. Binomial mixed model: additional details of statistical methods 13

S5 Appendix: Sensitivity analysis: comparison of primary binomial mixed model for HBsAg prevalence with i. a model that included all data (1992-2014) ii. Main model without the use of sampling weights 14

S6 Appendix. Sources of data for modelled transmission estimates 15

S7 Appendix. Summary of aggregate quality score domains for included studies 16

S8 Appendix. Quality score assessment for included studies 17

S9 Appendix: Summary of included prevalence surveys, stratified by WHO Africa sub-region 26

S10 Appendix. Details of included prevalence studies 27

S11 Appendix. Forest plot of HBsAg prevalence in pregnant women by WHO Africa sub-region Surveys taken from the 2014-2023 dataset and results stratified by WHO sub-region; [A] Eastern and Southern Africa; [B] Central Africa and [C] West Africa. Pooled estimates are from a binomial mixed model. 32

S12 Appendix. Plot of HBsAg prevalence stratified by quality score quartile 34

S13 Appendix. Meta-regression of characteristics associated with HBsAg prevalence in pregnant women attending for antenatal care 35

S14 Appendix. Forest plot of HBeAg prevalence in pregnant women, stratified by WHO Africa sub-region 36

S15 Appendix: Characteristics of included samples assessing mother to child transmission, stratified by vaccine status 37

S16 Appendix. Details of included MTCT studies 38

S17 Appendix. Forest plot of mother to child transmission rate, stratified by intervention sub-groups: [A] Birth dose; [B] Delayed birth dose; [C] HepB3 without a birth dose; [D] No vaccination; [E] Received maternal antiviral prophylaxis 41

S18 Appendix. Mother to child transmission stratified by vaccination subgroups, excluding cohorts which offered maternal antiviral prophylaxis: [A] Birth dose; [B] Delayed birth dose; [C] HepB3 without a birth dose; [D] No vaccination 43

S1 Appendix: Systematic review protocol

Review title: Vertical transmission of hepatitis B in the World Health Organisation African region: systematic review and meta-analysis

Anticipated or actual start date: 30/11/22

Anticipated completion date: 31/05/24

Stage of review at time of this protocol: Review stage Started

Preliminary searches Yes; Piloting of the study selection process Yes; Formal screening of search results against eligibility criteria Yes; Data extraction No; Risk of bias (quality) assessment No ; Data analysis No

Named contact: Nicholas Riches

Named contact email: Nicholas.riches@lstmed.ac.uk

Named contact address: Department of Clinical Sciences and International Public Health, Liverpool School of Tropical Medicine, Pembroke Place, Liverpool, L3 5QA

Named contact phone number: +44(0)1517053100

Organisational affiliation of the review: Liverpool School of Tropical Medicine; University of Liverpool; Malawi-Liverpool-Wellcome Programme, Blantyre, Malawi

Organisation web address:

https://www.lstmed.ac.uk/research/departments/international-public-health

https://www.liverpool.ac.uk/

https://www.mlw.mw/

Review team members and their organisational affiliations:

Nicholas Riches MBChB[1,2], Marc Y.R. Henrion PhD[2,3], Peter MacPherson PhD[4,5], Camilla Hahn MD[6], Rabson Kachala MD[7], Thomas Mitchell MBChB[2], Daniel Murray MD[2], Wongani Mzumara MPH[7], Owen Nkoka PhD[1,4], Alison J. Price PhD[1,5], Jennifer Riches MD[1], Aoife Seery MD[8], Noel Thom MD[1], Anne Loarec MD[9], Maud Lemoine PhD[10,11], Gibril Ndow PhD[10,11], Yusuke Shimakawa PhD[12], Peyton Thompson MD[13], Camille Morgan PhD[13], Shalini Desai PhD[14], Philippa Easterbrook PhD[14], Alexander J. Stockdale PhD[15,16]

1 Malawi Epidemiology and Intervention Research Unit, Lilongwe, Malawi

2 Department of Clinical Sciences, Liverpool School of Tropical Medicine, Liverpool, United Kingdom (UK)

3 Statistical Support Unit, Malawi Liverpool Wellcome Programme, Blantyre, Malawi

4 School of Health & Wellbeing, University of Glasgow, Glasgow, UK

5 Clinical Research Department, London School of Hygiene & Tropical Medicine, London, UK

6 Institute of Tropical Medicine, University of Tübingen, Tübingen, Germany

7 Viral Hepatitis Programme, Department of HIV/AIDS, Ministry of Health, Lilongwe, Malawi

8 East Sussex Healthcare NHS Trust, Bexhill, UK

9 Médecins Sans Frontières, Maputo, Mozambique

10 Department of Metabolism, Digestion and Reproduction, Imperial College London, London, UK

11 MRC Unit The Gambia, London School of Hygiene & Tropical Medicine, Banjul, The Gambia

12 Insitut Pasteur, Université Paris Cité, Unité d'Épidémiologie des Maladies Émergentes, Paris, France

13 Department of Pediatrics, Division of Infectious Diseases, University of North Carolina at Chapel Hill, North Carolina, USA

14 Global HIV, Hepatitis and STI Programme, World Health Organisation, Geneva, Switzerland

15 Department of Clinical Infection, Microbiology and Immunology, Institute of Infection, Veterinary and Ecological Sciences, University of Liverpool, Liverpool, UK

16 Malawi Liverpool Wellcome Programme, Blantyre, Malawi

Funding sources/sponsors: Nicholas Riches is supported by a Wellcome Trust Clinical PhD Fellowship. Wellcome Trust award number 203919_Z_16_Z

Conflicts of interest: YS has received a research grant from Gilead and research materials from Abbott Laboratories and Fujirebio Inc. The remaining authors report no conflicts of interest.

Review questions:

1. What is the prevalence of hepatitis B surface antigen (HBsAg) among pregnant women in the WHO African region, stratified by WHO African sub-regions?
2. What is the prevalence of HBsAg among infants of mothers with hepatitis B in the WHO African region, stratified by WHO African sub-regions, reflecting vertical transmission?
3. What is the trend in HBsAg prevalence in pregnant women and HBV-exposed infants in the WHO African region between 1992 and 2022?
4. What is the trend in vertical transmission events in the WHO African region, stratified by vaccination subgroup?

Searches:

We will search the following electronic bibliographic databases: PubMed, Scopus, EMBASE, Africa Index Medicus and African Journals Online. No language restrictions will be applied. The search will be limited to articles published between 1st January 1992 and 7th January 2024**.**

PUBMED (https://pubmed.ncbi.nlm.nih.gov/) Limit >1992

(hepatitis B[MeSH] OR hepatitis b[tiab] OR HBsAg[tiab] OR hepatitis b surface antigens[MeSH] or “hepatitis B surface antigen”[tiab])
(Africa[MeSH] OR Africa South of the Sahara[MeSH] or Africa[tiab] OR Angola[tiab] OR Benin[tiab] OR Botswana[tiab] OR "Burkina Faso"[tiab] OR Burundi[tiab] OR Cameroon[tiab] OR "Cape Verde"[tiab] OR "Central African Republic"[tiab] OR Chad[tiab] OR Comoros[tiab] OR Congo[tiab] OR Djibouti[tiab] OR "Equatorial Guinea"[tiab] OR Eritrea[tiab] OR Eswatini[tiab] OR Ethiopia[tiab] OR Gabon[tiab] OR Gambia[tiab] OR Ghana[tiab] OR Guinea[tiab] OR "Guinea Bissau"[tiab] OR "Ivory Coast"[tiab] OR "Cote d’Ivoire"[tiab] OR Kenya[tiab] OR Lesotho[tiab] OR Liberia[tiab] OR Madagascar[tiab] OR Malawi[tiab] OR Mali[tiab] OR Mauritania[tiab] OR Mauritius[tiab] OR Mozambique[tiab] OR Mocambique[tiab] OR Namibia[tiab] OR Niger[tiab] OR Nigeria[tiab] OR Principe[tiab] OR Reunion[tiab] OR Rwanda[tiab] OR "Sao Tome"[tiab] OR Senegal[tiab] OR Seychelles[tiab] OR "Sierra Leone"[tiab] OR Somalia[tiab] OR "South Africa"[tiab] OR “South Sudan”[tiab] OR Sudan[tiab] OR Swaziland[tiab] OR Tanzania[tiab] OR Togo[tiab] OR Tunisia[tiab] OR Uganda[tiab] OR Zambia[tiab] OR Zimbabwe[tiab])

EMBASE (https://pubmed.ncbi.nlm.nih.gov/)
Limit >1992

hepatitis b.mp. or exp hepatitis B/ or hepatitis b surface antigen.mp. or exp hepatitis B surface antigen/ or hbsag.mp
(africa or angola or benin or botswana or "Burkina Faso" or burundi or cameroon or "Cape Verde" or "Central African Republic" or chad or comoros or congo or djibouti or "Equatorial Guinea" or eritrea or ethiopia or eswatini or gabon or gambia or ghana or guinea or "Guinea Bissau" or "Ivory Coast" or "Cote d'Ivoire" or kenya or lesotho or liberia or madagascar or malawi or mali or mauritania or mauritius or mozambique or mocambique or namibia or niger or nigeria or principe or reunion or rwanda or "Sao Tome" or senegal or seychelles or "Sierra Leone" or somalia or "South Africa" or "sub-Saharan Africa" or sudan or "South Sudan" or swaziland or tanzania or togo or tunisia or uganda or zambia or zimbabwe).mp or sub-Saharan Africa.mp. or exp "Africa south of the Sahara"/

SCOPUS (https://www.scopus.com/search/form.uri?)
Limits: 1992-
Limits: Medicine/ Immunology/ Biochemsitry/ Multidisciplinary/ Health Professions/ Pharmacology

(TITLE-ABS-KEY (africa OR angola OR benin OR botswana OR "Burkina Faso" OR burundi OR cameroon OR "Cape Verde" OR "Central African Republic" OR chad OR comoros OR congo OR djibouti OR "Equatorial Guinea" OR eritrea OR ethiopia OR eswatini OR gabon OR gambia OR ghana OR guinea OR "Guinea Bissau" OR "Ivory Coast" OR "Cote d'Ivoire" OR kenya OR lesotho OR liberia OR madagascar OR malawi OR mali OR mauritania OR mauritius OR mozambique OR mocambique OR namibia OR niger OR nigeria OR principe OR reunion OR rwanda OR "Sao Tome" OR senegal OR seychelles OR "Sierra Leone" OR somalia OR "South Africa" OR "sub-Saharan Africa" OR sudan OR "South Sudan" OR swaziland OR tanzania OR togo OR tunisia OR uganda OR zambia OR zimbabwe)) AND (TITLE-ABS-KEY ("hepatitis B" OR hbsag OR "hepatitis B surface antigen")) AND PUBYEAR > 1991 AND PUBYEAR < 2023 AND (LIMIT-TO (SUBJAREA , "PHAR") OR LIMIT-TO (SUBJAREA , "MEDI") OR LIMIT-TO (SUBJAREA , "IMMU") OR LIMIT-TO (SUBJAREA , "BIOC") OR LIMIT-TO (SUBJAREA , "HEAL"))
AND (TITLE-ABS-KEY ("hepatitis b" OR hbv OR hbsag))

Africa Index Medicus (https://indexmedicus.afro.who.int/)
Limit: 1992-

Title/ Abstract/ Subject: "hepatitis B surface antigen" OR "HBsAg" OR "hepatitis B"

Africa Journals Online (https://www.ajol.info/index.php/ajol)
Searched using Google Scholar (https://scholar.google.com/)

site:ajol.info AND (HBsAg or "Hepatitis B surface antigen" or "hepatitis B")

Condition or domain being studied: chronic hepatitis B

Participants/population:

Inclusion:

Pregnant women and infants living in the WHO African region who have been tested for hepatitis B surface antigen using a validated diagnostic test.

Studies should include details of the population location, sampling method, and describe a representative sampling framework (random, consecutive recruitment over a specified interval, or a complete-recruitment strategy.

Exclusion:

The following studies will be excluded:

- Studies that do not define a random, consecutive, or complete testing sampling strategy
- Laboratory-based studies (without a sampling framework describing selection and recruitment procedures)
- Studies of acute hepatitis
- Basic science or animal studies
- Hospital-based studies selecting patients with acute medical, surgical or obstetric disease
- Studies that use a rapid diagnostic test that has not been WHO prequalified, Food and Drug Administration approved or CE marked as an in-vitro diagnostic test, or which has publicly available validation data demonstrating sensitivity >90% relative to an approved ELISA test within a similar population group within the WHO African region.

Intervention(s), exposure(s):

Diagnostic tests: Studies testing for hepatitis B surface antigen (HBsAg) using one or more of the following testing methods will be included:

- *Laboratory-based tests*: enzyme linked immunoassay (ELISA), chemiluminescent immunoassay (CLIA), electrochemical assay (ECA)
- *Rapid diagnostic tests (RDTs)*: Any RDT which meets any of the following criteria will be included in the review:
  - WHO pre-qualification
  - CE (conformité européenne) marked in-vitro diagnostic device
  - United States (US) Food and Drug Administration FDA approved, or meeting stringent regulatory approval (Japan, Australia, Canada, EU or USA)
  - Published evidence showing validation data of the RDT device vs. an approved assay in a comparable geographic and population setting fulfilling WHO prequalification criteria.

Studies with the following characteristics will be excluded:

- Study design: Systematic reviews (where lists of included studies will be reviewed for potential inclusion), case studies, and editorials will be excluded.
- Sampling method: Method not stated or insufficient detail to allow reproducibility. Non-random methods (except for consecutive sampling).

We will assess vertical transmission according to vaccination subgroup defined as:

- HepB3: Receipt of a 3-dose vaccination series, typically at 6, 10 and 14 weeks of infancy.
- Timely HepB-BD: Receipt of a birth dose hepatitis B vaccine delivered within 24 hours of life
- Delayed HepB-BD:Receipt of a birth dose hepatitis B vaccine delivered between 24 hours and 1 week of life
- Unvaccinated: No hepatitis B vaccination given
- Maternal antiviral prophylaxis: Antenatal antiviral treatment for mothers with tenofovir disoproxil fumarate

Comparator(s)/control: None

Types of study to be included: Observational or interventional, prospective or retrospective, cross-sectional, case-control and cohort studies.

Context: We will include community studies or hospital based studies.

Main outcome(s):

1. Pooled seroprevalence of HBsAg in pregnant women and infants born to HBV-positive mothers.
2. Rates of vertical transmission from HBsAg positive pregnant women, stratified by vaccine: unvaccinated, HepB3 commencing at 6 weeks, delayed birth dose provided between 24 hour and 1 week and timely birth dose within 24 hours of birth.

Additional outcome(s):

1. Prevalence of HBeAg positivity in pregnant women
2. HBV DNA estimation in pregnant women (categorised by above or below 200,000 IU/ml).

Data extraction (selection and coding):

Studies retrieved from the search strategy will be deduplicated and then have titles and abstracts screened by two independent reviewers. Studies marked for inclusion by either reviewer will then proceed to full text screening. Full text review will also be conducted in duplicate by two independent reviewers. Discrepant results will be identified and resolved through discussion by the reviewers.

Data will then be extracted from the list of included studies in duplicate using a cloud-based data extraction tool (implemented in google forms). Any discrepancies between each independently extracted data for each study will be resolved by an independent reviewer.

Risk of bias (quality) assessment: Quality assessment will be performed using a modified prevalence study assessment tool, which will use nine questions to assess the sampling framework, sampling procedures, response rate and the quality and completeness of diagnostic testing. (REF: *Munn Z, Moola S, Lisy K, Riitano D, Tufanaru C. Methodological guidance for systematic reviews of observational epidemiological studies reporting prevalence and incidence data. Int J Evid Based Healthc. 2015;13(3):147–153.)*

A principal component analysis will be used to estimate the overall risk of bias from these questions.

Strategy for data synthesis:

Describe the methods you plan to use to synthesise data. This must not be generic text but should be specific to your review and describe how the proposed approach will be applied to your data.

Assuming significant heterogeneity between study populations, we will estimate HBsAg prevalence with 95% confidence intervals using a binomial mixed model, with estimates weighted by the size of the population represented by the study (UN population estimates will be used for nationally-representative studies).

We will additionally as a sensitivity analysis consider the potential impact of the data quality score by adding the quality score as a variable in the binomial mixed model.

Forest plots will be made using the meta package in R. We will calculate I^2 to describe the proportion of variation attributable to heterogeneity, rather than sampling error.

We will describe the time trend in HBsAg prevalence in the WHO Africa region using linear regression models, adjusted for age, sex, study size, study quality.

We will conduct sensitivity analysis to investigate the effect of the type of study (e.g. sub-national vs. localised) included and the study quality (e.g. low vs. high study quality).

For assessment of the rate of vertical transmission we will use a generalised linear mixed model with random effects and logit transformation of transmission rates.

Analysis of subgroups or subsets:

Prevalence estimates will be stratified by:

1. Age
2. WHO African region subgroups:
    a. Central Africa 10 countries: Angola, Burundi, Cameroon, Central African Republic, Chad, Republic of Congo, Democratic Republic of the Congo, Equatorial Guinea, Gabon, and Sao Tome and Principe.
    b. Eastern and Southern Africa 20 countries: Botswana, Comoros, Eritrea, Ethiopia, Kenya, Lesotho, Madagascar, Malawi, Mauritius, Mozambique, Namibia, Rwanda, South Africa, Seychelles, South Sudan, Swaziland, Tanzania, Uganda, Zambia and Zimbabwe.
    c. West Africa 17 countries: Algeria, Benin, Burkina Faso, Cape Verde, Cote d'Ivoire, Gambia, Ghana, Guinea, Guinea Bissau, Liberia, Mali, Mauritania, Niger, Nigeria,

Subgroup analyses will depend on data availability.

Language: There will be no language restrictions applied, studies in languages unknown to the study team will be translated into English.

Keywords: Hepatitis B; pregnancy; vertical transmission; antenatal; prevalence; systematic review; meta-analysis; Africa

S2 Appendix. Details of HBsAg tests used

Author	Year of publication	HBsAg test type	HBsAg test name	Regulatory approval for RDTs
Aba, H.	2016	EIA/ELISA	Beijing Kinghawk Pharmaceutical Co., Ltd., China	
Abuku, V.	2023	RDT	Onsite HBsAg Rapid Test; CTK Biotech. San Diego, CA, USA	CE marked IVD
Adeyemi, A	2014	EIA/ELISA	Dia.Pro, Diagnositic Bioprobes Srl, Milan, Italy	
Adu-Sarkodie, Y.	1996	EIA/ELISA	Behring, Marburg, Germany	
Ahmed S.	1998	EIA/ELISA	Bioelisa, Barcelona, Spain	
Akani, C.	2005	EIA/ELISA	Not stated	
Andersson, M.	2013	EIA/ELISA	Abbott AxSYM, Abbott Diagnostics, Chicago, IL, USA	
Araya Mezgebo, T.	2018	EIA/ELISA	Monolisa HBsAg Ultra assay, Bio-Rad, France	
Arefaine, M.	2023	EIA/ELISA	Not stated	
Awole, M.	2005	RDT	Bioline HBsAG One Test, Abbott, USA	WHO PQ
Ayed, Z.	1995	EIA/ELISA	Auszyme Monoclonal diagnostic kit, Abbott, USA	
Bafa, T.	2020	EIA/ELISA	Not stated	
Balde,T.	2021	EIA/ELISA	DS-ELISA HBsAg; NPO Diagnostic Systems, Russia	
Bancha, B.	2020	RDT	Bioline HBsAg, Abbott, USA	WHO PQ
Bassey, E.	2009	EIA/ELISA	Monoclonal, ELISA, Abbot, USA	
Bayo, P.	2014	EIA/ELISA	Savyon Diagnostics Ltd, Ashod, Israel	
Bayu, H.	2020	EIA/ELISA	Dialab1 HBsAg, Dialab, Austria	
Bittaye, M.	2019	RDT	Determine, Abbott Laboratories, Chicago, USA	WHO PQ
Burnett, R.	2007	EIA/ELISA	IMxs assays HBsAg, Abbott Laboratories, Chicago, IL, USA	
Candotti, D.	2007	RDT	Determine, Abbott Laboratories, Chicago, USA	WHO PQ
Chotun, N.	2017	RDT	Determine, Alere Inc., MA, USA	WHO PQ
Croce, F.	2007	EIA/ELISA	Eti- Mak-4, DiaSorin, Vercelli, Italy	
Dabsu, R.	2018	RDT	SD Bioline, Abbott, USA	WHO PQ
Dagnew, M.	2022	EIA/ELISA	Linear Chemicals, SLU, Spain	
Dao, B.	2001	EIA/ELISA	Monolisa, Sanofi Pasteur Diagnoses, France	
De Paschale, M.	2014	EIA/ELISA	Hepanostika HBsAg Ultra, Marcy l'Etoile, France	
Deme, C.	2016	RDT	SD Bioline, Gyeonggi-do, South Korea	WHO PQ
Demeke, G.	2021	RDT	SD Bioline, Abbott, China	WHO PQ
Diale, Q.	2016	EIA/ELISA	Abbott Diagnostics, Germany	
Dionne-Odom, J.	2016	RDT	ABON, Alere, USA	CE marked IVD
Dortey, B.	2020	RDT	Onsite HBsAg Rapid Test; CTK Biotech, San Diego, CA, USA	CE marked IVD
Ducancelle, A.	2013	RDT	VIKIA HBs Ag, Biomerieux, Marcy l'Etoile, France	WHO PQ

Ekra, D.	2008	EIA/ELISA	Uni-form, Processor FlexTek - bioMeriex, Marcy l'Etoile, France	
Etti, M.	2021	CLIA	Not stated	
Firde, M.	2022	EIA/ELISA	Dialab HBsAg enzyme linked immunosorbent assay kit, Dialab GmbH, Austria	
Fofana, D.B.	2023	CLIA	ARCHITECT HBsAg, Abbott Diagnostics Division, USA	
Fomulu, N.	2013	EIA/ELISA	Monalisa HBsAg Ultra ELISA kit, BIO-RAD Laboratories, USA.	
Fowotade, A.	2021	EIA/ELISA	ELISA kit for HBsAg, Dia.Pro, Milan, Italy	
Gedefaw, G.	2019	EIA/ELISA	Hepanostika, Biomerieux, Boxtel, Netherland	
Geffert, K.	2020	RDT	SureScreen, Derby, United Kingdom	CE marked IVD
Genetu, K.	2022	RDT	SD Bioline, Yongin, South Korea	WHO PQ
Guingane, A.	2020	EIA/ELISA	Vidas Ultra, Biomerieux, France	
Harry, T.	1994	EIA/ELISA	Abbott, USA	
Helegbe, G.	2023	RDT	HBV surface antigen RDT kit; Premier Co., Ltd., Nagpur, India, and Transnational Technologies Inc., Manchester, UK	CE marked IVD
Ifeorah, I.	2017	EIA/ELISA	Enzyme-linked immunosorbent assay (ELISA) kits; Diagnostic Automation/Cortez Diagnostic, California, USA.	
Ikeme, A.	2006	EIA/ELISA	Not stated	
Ilboudo, D.	2002	EIA/ELISA	Not stated	
Itou-Ngaporo, A.	2016	EIA/ELISA	Bio-Rad Laboratories, Marnes La Coquette, France	
Joseph Davey, D.	2022	CLIA	Elecsys HBSAg II assay, Roche diagnostics, Switzerland	
Kassa, D.	2019	EIA/ELISA	Hepanostika ELISA kit, Biomerieux, Marcy l'Etoile, France.	
Kfutwah, A.	2012	EIA/ELISA	Monolisa AgHBs Plus Biorad, France.	
Kibassa, C.	2004	EIA/ELISA	Hepanostika HBsAg Uni-Form 11 test kit, Organon Teknika, Boxtel, Netherlands.	
Kirkbak, A.	2017	EIA/ELISA	Murex, Abbott, Italy	
Kolawole, O.	2012	EIA/ELISA	Biotech HBsAg, USA	
Komas, N.	2018	EIA/ELISA	Abbott-Murex, United Kingdom	
Koumba Mavoungou, D.	2023	EIA/ELISA	Monolisa TM HBsAg ULTRA; Bio-Rad, Marnes la Coquette, France	
Ladner, J.	1998	EIA/ELISA	EIA, Auszyme, Abbott Laboratories, Abbott Park, IL, USA.	
Loarec, A.	2022	RDT	Determine HBsAg 2, Abbott, USA	WHO PQ
Lohoues-Kouacou, M.	1998	EIA/ELISA	MONOLISA Ag HBs 2nd generation, Sanofi Pasteur, France	
MacLean, B.	2012	EIA/ELISA	D BIO, Standard Diagnostic, India	
Maiga, Y.	1992	EIA/ELISA	Auszyme, Abbott, USA	
Makuwa, M.	2008	EIA/ELISA	Monolisa Ag HBV-Plus, Biorad, Marnes la Coquette, France	
Mamadou, S.	2012	EIA/ELISA	Monolisa HBsAg Ultra, Bio-Rad, USA; ImmunoComb II HBsAg, Orgenics, Israel	
Mansour, W.	2012	EIA/ELISA	ELISA; Axsym-Abbott, Rungis, France	

Manyahi, J.	2017	EIA/ELISA	ARCHITECT ci4100 Integrated Immunoassay and Clinical Chemistry System, Abbott Laboratories, Abbott Park, IL, USA	
Marama, T.	2020	EIA/ELISA	Dialab GmbH, Wiener Neudorf, Austria	
Mavenyengwa, R.	2010	CLIA	Architect, Abbott, USA	
Meier-Stephenson, V.	2020	CLIA	Architect, Abbott, USA	
Menendez, C.	1999	EIA/ELISA	Ortho Diagnostics Systems, NJ, USA	
Metaferia, Y.	2016	EIA/ELISA	Dialab GmbH, Wiener Neudorf, Austria	
Mhata, P.	2017	CLIA	ARCHITECT Abbott Laboratories, Sligo, Ireland	
Morgan, C.	2023	RDT	Determine 2, Abbott	WHO PQ
Mugabiirwe, N.	2022	RDT	SD BIOLINE HBsAg, Abbott Diagnostics Korea Inc, Gihueng-gu, Yongin- si, Republic of Korea	WHO PQ
Mustapha, G.	2020	EIA/ELISA	LabACONR; Hangzhou Biotest Biotech Co., Ltd., China	
Mutagoma, M.	2017	CLIA	ARCHITECT, Abbott, USA	
Ndow, G.	2023	RDT	HBsAg RDT, Abbott, USA	WHO PQ
Ndumbe, P1.	1992	EIA/ELISA	Abbott Auszyme, USA	
Ndumbe P2.	1994	EIA/ELISA	Abbott Diagnostics, North Chicago, USA	
Ngaira, J.	2016	EIA/ELISA	Hepanostika, HBsAg Ultra ELISA kit, Biomerieux, Netherlands	
Nlinwe, N.	2021	RDT	OnSite HBsAg Rapid Test, CTK Biotech, Inc., USA	CE marked IVD
Oliveira, D.	2020	RDT	Laboquick HBsAg Test, Koroglu Medical Devices, Turkey	CE marked IVD
Oluremi, A.	2020	EIA/ELISA	HBsAg ELISA kit, Melsin Medical Co., Limited, China	
Ondigui, J.	2023	EIA/ELISA	Murex 168 HBsAg, Abbott, USA	
Onwere, S.	2012	EIA/ELISA	Acon laboratories, USA	
Osazuwa, F.	2012	EIA/ELISA	Clinotech Diagnostics, Canada	
Oshitani, H1	1996	EIA/ELISA	Frelisa HBsAg, Fujirebio, Tokyo, Japan	
Oshitani, H2	1995	EIA/ELISA	Frelisa HBsAg, Fujirebio, Tokyo, Japan	
Ouoba, S.	2023	RDT	Determine 2 HBsAg, Abbott, USA	WHO PQ
Peliganga, L.	2022	RDT	HBsAg Determine; Abbott, USA	WHO PQ
Pellizzer, G.	1994	EIA/ELISA	Abbott Laboratories, Chicago, IL, USA	
Ramos, M.	2011	EIA/ELISA	HBsAg II cobas, Roche Diagnostics, Mannheim, Germany.	
Rashid, S.	2014	EIA/ELISA	Microparticle enzyme immunoassay, Abbott AxSYM, Germany	
Roble, A.	2020	EIA/ELISA	Wantai HBV Diagnostic kit, China	
Roingeard, P.	1993	EIA/ELISA	Auszyme Monoclonal, Abbott, North Chicago, IL, USA	
Rouet, F.	2004	EIA/ELISA	Abbott Laboratories, Abbott Park, North Chicago, IL, USA	
Sangare, L.	2009	RDT	Determine HBsAg, Abbott, USA	WHO PQ
Shimakawa, Y.	2022	RDT	VIKIA HBsAg, BioMerieux, France	WHO PQ
Tamandjou, C.	2019	RDT	Determine HBsAg rapid test, Abbott, USA	WHO PQ

Tanjong, R.	2016	RDT	SD Bioline, Standard Diagnostics, USA	WHO PQ
Tegegne, D.	2014	EIA/ELISA	Dialab GmbH, Wiener Neudorf, Austria	
Thahir, S.	2023	RDT	Standard Q HBsAg RDT; SD Biosensor, Gyeonggi-do, Republic of Korea	WHO PQ
Thomson P.	2021	RDT	Alere Determine HBsAg, Abbott Diagnostics, Abbott Park, IL, USA	WHO PQ
Thumbiran, N.	2014	EIA/ELISA	Siemens Healthcare Diagnostics, NY, USA	
Torimiro, J.	2018	EIA/ELISA	Monolisa, Bio-Rad, France	
Ugbebor, O.	2011	EIA/ELISA	Clinotech Diagnostics, Canada	
Umare, A.	2016	EIA/ELISA	Dialab HBsAg ELISA kit; Dialab GmbH, Austria	
Umer, A.	2023	EIA/ELISA	Human type, Germany	
Utoo, B.	2013	EIA/ELISA	DIA-PRO Diagnostic Bioprobes Srl via columella, Italy	
Volker, F.	2017	EIA/ELISA	HBsAg(v2)/AXSYM Abbott, Germany and HBsAg EIA Test Kit Ascon, USA	
Vueba, A.	2021	EIA/ELISA	VIDAS, Biomerieux, Portugal	
Wakjira, M.	2022	EIA/ELISA	Not stated	
Woldesonbet, Z1.	2016	EIA/ELISA	Not stated	
Woldesonbet, Z2.	2016	EIA/ELISA	Not stated	
Yohanes, T.	2016	EIA/ELISA	Dialab GmbH, Wiener Neudorf, Austria	
Zenebe, Y.	2014	EIA/ELISA	Linear chemicals, Barcelona, Spain	

Abbreviations: CE marked IVD Conformité Européenne marked in vitro diagnostic device; CLIA chemiluminescent immunoassay; EIA/ELISA enzyme immunoassay/enzyme immunosorbant assay; RDT rapid diagnostic test; WHO PQ World Health Organization prequalified;

S3 Appendix. Quality assessment checklist

Adapted from Munn Z, Moola S, Lisy K, Riitano D, Tufanaru C. Chapter 5: systematic reviews of prevalence and incidence. JBI Manual for Evidence Synthesis. JBI 2020. Available at: https://jbi.global/sites/default/files/2021-10/Checklist_for_Prevalence_Studies.docx [Accessed 18th July 2024]

Domain	Description
a. Was the sampling framework appropriate to address the target population?	Is the sample representative of the target population? eg. avoided inappropriate exclusions, characteristics of the participants match the population of interest (for example consider socioeconomic bias from a private fee-paying provider if there is a public health system)
b. Were study participants sampled in an appropriate way? (to facilitate representative sampling of the population, description of how sampling was performed)	Representative sampling methods involve random probabilistic sampling from a define population of interest, or an attempt to recruit an entire eligible population over a given time/catchment population. Low representative samples include: convenience samples, a lack of a detailed sampling frame.
c. Are the sampling methods and eligibility criteria (inclusion and exclusion criteria) both clear and appropriate?	Are these reported in sufficient to reproduce this study?
d. Were the study subjects and the setting described in detail?	Sufficient detail should be provided to determine comparability to other cohorts: age distribution, HIV prevalence, gestational age at enrolment, clinic setting
e. Was the response rate described?	Is there a study flowchart or report of the number of refusals or losses at each stage of the survey/ sampling/ testing?
f. Was the response rate adequate?	If a high non-reponse rate (eg. >15%), have the authors performed an analysis of characteristics associated with response, especially considering socoioeconomic and demographic characteristics? Is non-response likely to be related to the outcome (HBsAg prevalence).

S4 Appendix. Binomial mixed model: additional details of statistical methods

As a sensitivity analysis, a binomial mixed model was developed to estimate the probability of HBsAg detection in pregnant women. Here we describe details of the statistical model and how prevalence estimates have been obtained.

Let Y_i denote the number of pregnant women who tested positive for HBsAg out of n_i for the i-th survey, for $i = 1, \ldots, n$.

We then assumed that, conditionally on independent and identically distributed Gaussian random effects Z_i with mean zero and variance σ^2, the Y_i were mutually independent random variables, distributed according to binomial distributions with probability of a positive HBsAg test p_i, such that:

$$log\left\{\frac{p_i}{1-p_i}\right\} = \beta_0 + \beta_1 + Z_i\,. \quad (1)$$

To estimate the unknown regression coefficients, β_0 and β_1, and random effects variance,σ^2, we then defined the likelihood function for $\boldsymbol{\theta} = (\beta_0, \beta_1, \sigma^2)$ as:

$$L(\theta) \;=\; \prod_{i=1}^{n} \quad L_i(\theta), \quad (2)$$

where

$$L_i(\theta) = \int_{-\infty}^{+\infty} \quad [Z_i][Y_i \mid Z_i]\, dZ_i \quad (3)$$

Finally, we maximised (2) by approximating the integral in (3) with a quasi Monte Carlo method.

Let C denote the set of surveys falling within a predefined geographical area (e.g. WHO region). To predict HBsAg prevalence among pregnant women for a specific WHO region, we took B = 10,000 samples, $p_i^{(j)}$, j=1, …, B, from the distribution of p_i conditioned on the data, also known as the predictive distribution for p_i, to obtain, (4)

$$\hat{p}_C = \frac{1}{B}\sum_{j=1}^{B}\frac{\sum_{i\in C} w_i p_i^{(j)}}{\sum_{i\in C} w_i} \quad (4)$$

where w_i is a weight representing the number of discrete geographical sites sampled by each study. A single centre study was given 1 point. Where a study sampled from multiple locations within one geographical area (e.g. a city), this was given 1.5 points. Where studies had multiple discrete and independent antenatal clinic locations the points for each location were added to a maximum total of 5 points. This procedure provided additional weighting to studies presenting data in aggregate form from multiple discrete underlying populations, with a maximum weighting applied to prevent excessive influence at the regional level from large studies from individual countries.

S5 Appendix: Sensitivity analysis: comparison of primary binomial mixed model for HBsAg prevalence with i. a model that included all data (1992-2014) ii. Main model without the use of sampling weights

Region	Primary model, % [95% confidence interval]	Binomial mixed model described in Appendix 4 with sampling weights for geographic coverage: data from 2014-2023, % [95% confidence interval]	Binomial mixed model described in Appendix 4 with sampling weights for geographic coverage across entire data range: data from 1992-2023, % [95% confidence interval]	Binomial mixed model with country-level estimates weighted by proportion of women of childbearing age in WHO sub-region to provide regional and sub-regional estimates: data from 2014-2023, % [95% confidence interval]
WHO African Region	6.2 [5.3 – 7.2]	6.3 [6.0 -6.5]	6.9 [6.7-7.2]	5.88 (5.54-6.20)
Central Africa	7.0 [4.9 – 10.0]	7.4 [6.9 – 7.8]	8.4 [8.0-8.9]	4.95 (4.53-5.41)
East and Southern Africa	4.4 [3.6 -5.4]	5.5 [5.3 – 5.8]	5.4 [5.1-5.6]	7.77 (6.77-8.91)
West Africa	7.5 [5.9 -9.4]	7.1 [6.5 – 7.7]	8.7 [8.2 – 9.1]	6.06 (5.66-6.55)

S6 Appendix. Sources of data for modelled transmission estimates

Indicator	Source	Link
HepB birth dose: The percentage in the target population who received HepB birth dose within the first 24 hours of birth in a given year.	WHO/UNICEF Estimates of National Immunization Coverage (WUENIC)	https://immunizationdata.who.int/pages/coverage/HEPB.html
HepB3: The percentage in the target population who have received three doses of Hepatitis B containing vaccine in a given year.	WHO/UNICEF Estimates of National Immunization Coverage (WUENIC)	https://immunizationdata.who.int/pages/coverage/HEPB.html
Total live births per year	United Nations, Department of Economic and Social Affairs, Population Division (2022). World Population Prospects.	https://population.un.org/wpp/
Female population of reproductive age (15-49 years)	United Nations, Department of Economic and Social Affairs, Population Division (2022). World Population Prospects.	https://population.un.org/wpp/
Antenatal care 1+ visit - percentage of women (aged 15-49 years) attended at least once during pregnancy by skilled health personnel	UNICEF data warehouse: Maternal, child and newborn health	https://data.unicef.org/resources/data_explorer/unicef_f/
Institutional deliveries - percentage of deliveries in a health facility	UNICEF data warehouse: Maternal, child and newborn health	https://data.unicef.org/resources/data_explorer/unicef_f/

S7 Appendix. Summary of aggregate quality score domains for included studies

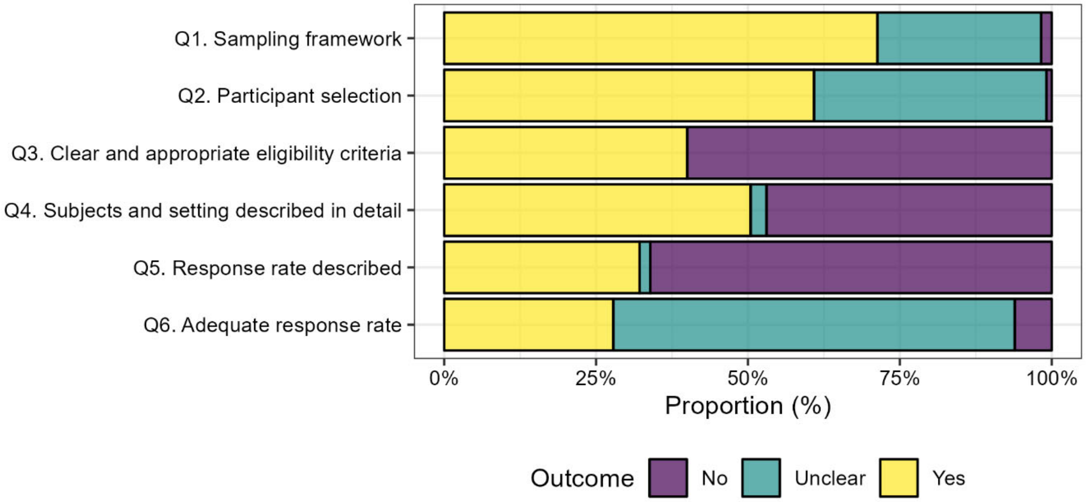

S8 Appendix. Quality score assessment for included studies

Study	Was the sampling framework appropriate to address the target population?	Were study participants sampled in an appropriate way?	Are the sampling methods and eligibility criteria (inclusion and exclusion criteria) both clear and appropriate?	Were the study subjects and the setting described in detail?	Was the response rate described?	Was the response rate adequate?	Quality score
**Aba, H., 2016**	Yes	Unclear	No	No	No	Unclear	-1.63
**Abuku, V., 2023**	Yes	Yes	Yes	Yes	Yes	Yes	2.84
**Adeyemi, A., 2014**	Yes	Yes	No	No	No	Unclear	-0.91
**Adu-Sarkodie, Y., 1996**	Unclear	Unclear	No	No	No	Unclear	-2.22
**Ahmed S., 1998**	Yes	Unclear	No	Yes	Yes	No	0.19
**Akani, C., 2005**	Yes	Yes	No	No	No	Unclear	-0.91
**Andersson, M., 2013**	Unclear	No	Yes	Yes	Yes	Yes	1.53
**Araya Mezgebo, T., 2018**	Unclear	Unclear	No	Yes	No	Unclear	-1.47
**Arefaine, M., 2023**	Yes	Unclear	No	No	No	Unclear	-1.63
**Awole, M., 2005**	Yes	Unclear	No	No	No	Unclear	-1.63
**Ayed, Z, 1995**	Yes	Unclear	No	No	No	Unclear	-1.63
**Bafa, T., 2020**	Yes	Yes	Yes	Yes	Yes	Yes	2.84

S8 Appendix. Quality score assessment for included studies

Study	Was the sampling framework appropriate to address the target population?	Were study participants sampled in an appropriate way?	Are the sampling methods and eligibility criteria (inclusion and exclusion criteria) both clear and appropriate?	Were the study subjects and the setting described in detail?	Was the response rate described?	Was the response rate adequate?	Quality score
**Balde,T., 2021**	Unclear	Unclear	No	No	No	Unclear	-2.22
**Bancha, B., 2020**	Yes	Yes	Yes	No	Yes	Yes	2.09
**Bassey, E., 2009**	Yes	Unclear	No	Yes	No	Unclear	-0.88
**Bayo, P., 2014**	Yes	Yes	Yes	Yes	Yes	Yes	2.84
**Bittaye, M., 2019**	Yes	Yes	Yes	Yes	No	Unclear	0.65
**Burnett, R., 2007**	Yes	Unclear	No	No	Yes	Yes	0.56
**Candotti, D., 2007**	Unclear	Unclear	No	No	No	Yes	-1.10
**Chotun, N., 2017**	Yes	Yes	Yes	Yes	Yes	Yes	2.84
**Croce, F., 2007**	Yes	Yes	No	No	No	Unclear	-0.91
**Dabsu, R., 2018**	Yes	Yes	No	Yes	Yes	Yes	2.04
**Dagnew, M., 2022**	Yes	Unclear	Yes	No	No	Unclear	-0.83
**Dao, B., 2001**	Unclear	Unclear	No	Yes	No	Unclear	-1.47
**De Paschale, M., 2014**	Yes	Unclear	No	Yes	No	Unclear	-0.88

S8 Appendix. Quality score assessment for included studies

Study	Was the sampling framework appropriate to address the target population?	Were study participants sampled in an appropriate way?	Are the sampling methods and eligibility criteria (inclusion and exclusion criteria) both clear and appropriate?	Were the study subjects and the setting described in detail?	Was the response rate described?	Was the response rate adequate?	Quality score
**Deme, C., 2016**	Unclear	Yes	No	Yes	No	Unclear	-0.74
**Demeke, G., 2021**	Yes	Yes	Yes	Yes	No	Unclear	0.65
**Diale, Q., 2016**	Yes	Yes	Yes	Yes	Yes	Yes	2.84
**Dionne-Odom, J., 2016**	Unclear	Unclear	No	Yes	No	Unclear	-1.47
**Dortey, B., 2020**	Yes	Yes	Yes	Yes	Yes	Yes	2.84
**Ducancelle, A., 2013**	Unclear	Yes	No	No	No	Unclear	-1.49
**Ekra, D., 2008**	Yes	Unclear	Yes	Yes	Yes	Yes	2.11
**Etti, M., 2021**	Unclear	Unclear	No	No	No	Unclear	-2.22
**Firde, M., 2022**	Unclear	Yes	No	No	Yes	Yes	0.70
**Fomulu, N., 2013**	Yes	Yes	Yes	Yes	Yes	No	1.72
**Fowotade, A., 2021**	Yes	Yes	Yes	Yes	No	Unclear	0.65
**Gedefaw, G., 2019**	Yes	Yes	No	Yes	Yes	Yes	2.04
**Geffert, K., 2020**	Yes	Yes	No	Yes	No	Unclear	-0.15

S8 Appendix. Quality score assessment for included studies

Study	Was the sampling framework appropriate to address the target population?	Were study participants sampled in an appropriate way?	Are the sampling methods and eligibility criteria (inclusion and exclusion criteria) both clear and appropriate?	Were the study subjects and the setting described in detail?	Was the response rate described?	Was the response rate adequate?	Quality score
**Genetu, K., 2022**	Yes	Yes	No	Yes	Yes	Yes	2.04
**Guidozzi, F.B., 1992**	Unclear	Yes	No	No	No	Unclear	-1.49
**Guingane, A., 2020**	Yes	Yes	Yes	No	Yes	No	0.96
**Harry, TO., 1994**	Unclear	Yes	Yes	No	No	Unclear	-0.69
**Helegbe, G., 2023**	Yes	Yes	Yes	Yes	Yes	Yes	2.84
**Ifeorah, I., 2017**	Yes	Unclear	No	No	No	Unclear	-1.63
**Ikeme, A., 2006**	Yes	Yes	No	No	No	Unclear	-0.91
**Ilboudo, D., 2002**	No	Unclear	Yes	No	No	Unclear	-1.42
**Itou-Ngaporo, A., 2016**	Yes	Unclear	No	No	No	Unclear	-1.63
**Joseph Davey, D., 2022**	Yes	Unclear	Yes	Yes	No	Unclear	-0.08
**Kassa, D., 2019**	Unclear	Unclear	No	No	No	Unclear	-2.22
**Kfutwah, A., 2012**	Yes	Yes	No	Yes	No	Unclear	-0.15
**Kibassa, C., 2004**	Yes	Yes	Yes	Yes	No	Unclear	0.65

S8 Appendix. Quality score assessment for included studies

Study	Was the sampling framework appropriate to address the target population?	Were study participants sampled in an appropriate way?	Are the sampling methods and eligibility criteria (inclusion and exclusion criteria) both clear and appropriate?	Were the study subjects and the setting described in detail?	Was the response rate described?	Was the response rate adequate?	Quality score
**Kirkbak, A., 2017**	Yes	Yes	Yes	Yes	No	Unclear	0.65
**Kolawole, O., 2012**	Yes	Unclear	No	No	No	Unclear	-1.63
**Komas, N., 2018**	Yes	Yes	Yes	No	No	Unclear	-0.10
**Koumba Mavoungou, D., 2023**	Yes	Yes	Yes	No	Unclear	Unclear	-0.10
**Ladner, J., 1998**	Yes	Yes	Yes	No	No	Unclear	-0.10
**Loarec, A., 2022**	Yes	Yes	Yes	Yes	No	Unclear	0.65
**Lohoues-Kouacou, M., 1998**	Yes	Yes	No	No	No	Unclear	-0.91
**MacLean,B., 2012**	Yes	Yes	Yes	No	No	Unclear	-0.10
**Maiga, Y., 1992**	Yes	Unclear	No	No	No	Unclear	-1.63
**Makuwa, M., 2008**	Unclear	Unclear	No	No	No	Unclear	-2.22
**Mamadou, S., 2012**	Unclear	Yes	No	Yes	Yes	Yes	1.45
**Mansour, W., 2012**	Yes	Unclear	No	No	No	Unclear	-1.63

S8 Appendix. Quality score assessment for included studies

Study	Was the sampling framework appropriate to address the target population?	Were study participants sampled in an appropriate way?	Are the sampling methods and eligibility criteria (inclusion and exclusion criteria) both clear and appropriate?	Were the study subjects and the setting described in detail?	Was the response rate described?	Was the response rate adequate?	Quality score
**Manyahi, J., 2017**	Yes	Yes	Yes	Yes	No	Unclear	0.65
**Marama, T., 2020**	Yes	Yes	Yes	No	Yes	Yes	2.09
**Mavenyengwa, R., 2010**	Unclear	Yes	Yes	Yes	Yes	Yes	2.25
**Meier-Stephenson, V., 2020**	Yes	Unclear	No	Yes	No	Unclear	-0.88
**Menendez, C., 1999**	Yes	Yes	Yes	Yes	No	Unclear	0.65
**Metaferia, Y., 2016**	Yes	Yes	Yes	Yes	Yes	Yes	2.84
**Mhata, P., 2017**	Yes	Unclear	No	Yes	Yes	No	0.19
**Morgan, C., 2023**	Unclear	Unclear	No	No	No	Unclear	-2.22
**Mugabiirwe, N., 2022**	Yes	Yes	No	Yes	No	Unclear	-0.15
**Mustapha, G., 2020**	Yes	Yes	No	No	No	Unclear	-0.91
**Mutagoma, M., 2017**	Yes	Yes	Yes	Yes	Yes	Yes	2.84
**Ndow, G., 2023**	Yes	Yes	No	No	Yes	Yes	1.28

S8 Appendix. Quality score assessment for included studies

Study	Was the sampling framework appropriate to address the target population?	Were study participants sampled in an appropriate way?	Are the sampling methods and eligibility criteria (inclusion and exclusion criteria) both clear and appropriate?	Were the study subjects and the setting described in detail?	Was the response rate described?	Was the response rate adequate?	Quality score
**Ndumbe, P., 1992**	Yes	Unclear	No	No	No	Unclear	-1.63
**Ndumbe, P., 1994**	Unclear	Yes	No	Yes	Yes	Yes	1.45
**Ngaira, J., 2016**	Unclear	Yes	Yes	Unclear	No	Unclear	-0.69
**Nlinwe, N., 2021**	Yes	Yes	Yes	No	Yes	Yes	2.09
**Oliveira, D., 2020**	Yes	Yes	No	Yes	No	Unclear	-0.15
**Oluremi, A., 2020**	Yes	Unclear	Yes	No	No	Unclear	-0.83
**Ondigui, J., 2023**	Unclear	Yes	No	No	No	Unclear	-1.49
**Onwere, S., 2012**	Unclear	Yes	No	Yes	No	Unclear	-0.74
**Osazuwa, F., 2012**	Yes	Yes	No	No	Yes	No	0.16
**Oshitani, H., 1996**	Yes	Unclear	No	No	No	Unclear	-1.63
**Oshitani, H., 1995**	Yes	Yes	No	No	No	Unclear	-0.91
**Ouoba, S., 2023**	Yes	Yes	Yes	Yes	Yes	Yes	2.84
**Peliganga, L., 2022**	Unclear	Unclear	No	Yes	No	Unclear	-1.47

S8 Appendix. Quality score assessment for included studies

Study	Was the sampling framework appropriate to address the target population?	Were study participants sampled in an appropriate way?	Are the sampling methods and eligibility criteria (inclusion and exclusion criteria) both clear and appropriate?	Were the study subjects and the setting described in detail?	Was the response rate described?	Was the response rate adequate?	Quality score
**Pellizzer, G., 1994**	Unclear	Yes	No	No	No	Unclear	-1.49
**Qolohle, D., 1995**	Yes	Yes	No	Yes	Yes	Yes	2.04
**Ramos, M., 2011**	Yes	Yes	No	Yes	No	Unclear	-0.15
**Rashid, S., 2014**	Yes	Yes	No	Yes	No	Unclear	-0.15
**Roble, A., 2020**	Yes	Yes	Yes	Yes	Yes	Yes	2.84
**Rouet, F., 2004**	Unclear	Yes	No	No	No	Unclear	-1.49
**Sangare, L., 2009**	Yes	Unclear	Yes	Yes	Yes	Yes	2.11
**Shimakawa, Y., 2022**	Yes	Yes	Yes	Yes	Yes	No	1.72
**Tamandjou, C., 2019**	Unclear	Yes	No	No	No	Unclear	-1.49
**Tanjong, R., 2016**	Yes	Yes	No	Yes	Yes	Yes	2.04
**Tegegne, D., 2014**	Yes	Yes	No	Yes	No	Unclear	-0.15
**Thahir, S., 2023**	Yes	Unclear	Yes	No	No	Unclear	-0.83
**Thomson P., 2021**	Yes	Yes	Yes	Yes	Yes	Yes	2.84

S8 Appendix. Quality score assessment for included studies

Study	Was the sampling framework appropriate to address the target population?	Were study participants sampled in an appropriate way?	Are the sampling methods and eligibility criteria (inclusion and exclusion criteria) both clear and appropriate?	Were the study subjects and the setting described in detail?	Was the response rate described?	Was the response rate adequate?	Quality score
**Thumbiran, N., 2014**	Unclear	Unclear	No	No	No	Unclear	-2.22
**Torimiro, J., 2018**	Yes	Unclear	No	Yes	No	Unclear	-0.88
**Ugbebor, O., 2011**	Yes	Unclear	No	No	No	Unclear	-1.63
**Umare, A., 2016**	Yes	Yes	Yes	Yes	Yes	Yes	2.84
**Umer, A., 2023**	Yes	Yes	Yes	Yes	Yes	Yes	2.84
**Utoo, B., 2013**	Yes	Unclear	No	No	No	Unclear	-1.63
**Volker, F., 2017**	Unclear	Unclear	No	Yes	Yes	No	-0.40
**Vueba, A., 2021**	Yes	Yes	No	Yes	No	Unclear	-0.15
**Wakjira, M., 2022**	Yes	Yes	Yes	No	No	Unclear	-0.10
**Woldesonbet, Z1, 2016**	Unclear	Unclear	No	No	No	Unclear	-2.22
**Woldesonbet, Z2., 2016**	Unclear	Unclear	No	No	No	Unclear	-2.22
**Yohanes, T., 2016**	Yes	Yes	No	No	No	Unclear	-0.91
**Zenebe, Y. , 2014**	Yes	Yes	Yes	Yes	No	Unclear	0.65

S9 Appendix: Summary of included prevalence surveys, stratified by WHO Africa sub-region

Characteristic	Central Africa	Eastern and Southern Africa	West Africa	Overall
**Number of surveys**	20	54	39	113
**Countries included**	Angola, Cameroon, Central African Republic, Democratic Republic of Congo, Gabon, Republic of the Congo,	Ethiopia, Kenya, Malawi, Mozambique, Namibia, Rwanda, South Africa, South Sudan, Tanzania, Uganda, Zambia, Zimbabwe	Algeria, Benin, Burkina Faso, Gambia, Ghana, Guinea, Ivory Coast, Mali, Mauritania, Niger, Nigeria	
**Total number of pregnant women tested for HBsAg**	48,976	82,822	59,185	190,983
**Number tested per study** (median; IQR)	930 (429-1,448)	408 (282-1,044)	515 (302-1,020)	498 (299-1,186)
**Age of participants** (median of mean ages; IQR)	26·7 (25·4, 27·6)	26·0 (25·6, 27·2)	26·9 (24·8, 27·1)	26·1 (25·1-27·5)
Not reported	10	33	28	71
**HBsAg test type** (n, %)				
ELISA/EIA	11 (55·0%)	36 (66·7%)	31 (79·5%)	78 (69·0%)
CLIA	0 (0%)	5 (9·3%)	0 (0%)	5(4·4%)
RDT	9 (45·0%)	13 (24·1%)	8 (20·5%)	30 (26·5%)
**HIV prevalence** (%, median; IQR)	3·5% (0·8-8·4%)	5·1% (0·0-9·3%)	2·0% (0·7-4·2%)	3·5% (0·6-7·4%)
Not reported	9	24	22	55
**HBeAg prevalence reported (among HBsAg positive women)**	13 (65·0%)	14 (25·6%)	15 (38·5%)	42 (37·2%)
**Median year of national vaccine introduction among included studies** (range)	2005 (2003-2006)	2007 (1994-2014)	2004 (1995-2008)	2004 (1994-2014)
**Years between vaccine introduction and study mid-point** (median; IQR)	8·2 (4·0-12·3)	9·0 (5·0-13·3)	6·7 (-1.2 -13·3)	8·2 (1·8-13·4)

Abbreviations: CLIA: Chemiluminescence assay; EIA: Enzyme immunoassay; ELISA: Enzyme-linked immunoassay; IQR: Interquartile range; RDT: Rapid diagnostic test

S10 Appendix. Details of included prevalence studies

Author	Country	WHO region	Location	Year of publication	Study start year	Study stop year
Aba, H.O	Nigeria	West Africa	Kaduna Metropolis<br>44 Nigerian Army Reference Hospital<br>St. Gerard Hospital<br>Barau Dikko Specialist Hospital<br>Dantsoho Memorial Hospital	2016	2011	2011
Adeyemi, A	Nigeria	West Africa	University College Hospital, Ibadan<br>Idi- Ogungun Health Centre<br>Adeoyo maternity Hospital.	2014	2011	2011
Andersson, M. I.	South Africa	Eastern and Southern Africa	Western Cape Province	2013	2008	2008
Araya Mezgebo, T	Ethiopia	Eastern and Southern Africa	SE zones of Tigray regional state, capital city Mekelle	2018	2015	2015
Bafa, TA.	Ethiopia	Eastern and Southern Africa	Atat Hospital, Azer	2020	2017	2017
Balde,T	Guinea	West Africa	Conakry	2021	NA	NA
Bancha, B.	Ethiopia	Eastern and Southern Africa	Wolaita Zone	2020	2018	2018
Bayo, P.	Uganda	Eastern and Southern Africa	Lacor Referral Hospital, Gulu Referral Hospital, Gulu District	2014	2012	2013
Bittaye, M.	Gambia	West Africa	Edward Francis Small Teaching Hospital, Banjul	2019	2015	2015
Chotun, N.	South Africa	Eastern and Southern Africa	Tygerberg Hospital, Cape Town	2017	2014	2014
Dabsu, R.	Ethiopia	Eastern and Southern Africa	Nekemte Referral Hospital<br>Nekemte Health Center<br>Getema Health Center<br>Arjo Gudetu and Sire Health Center<br>All in East Wollega Zone, West Oromia	2018	2014	2014
Dagnew, M.	Ethiopia	Eastern and Southern Africa	Amhara Regional State	2022	2018	2019
De Paschale, M.	Benin	West Africa	Saint Jean de Dieu de Tanguieta hospital, Atacora district	2014	2011	2011

Deme, C.	Ethiopia	Eastern and Southern Africa	Gambo Rural Hospital	2016	2011	2012
Demeke, G.	Ethiopia	Eastern and Southern Africa	Debre Markos Referral Hospital	2021	2017	2017
Diale, Q.	South Africa	Eastern and Southern Africa	1 Military Hospital, Tshwane	2016	2008	2013
Dionne-Odom, J2	Cameroon	Central Africa	Banso Baptist Hospital<br>Mbingo Baptist Hospital<br>Baptist Hospital Mutengene<br>Mboppi Baptist Hospital in Douala.	2016	2014	2014
Dortey, B.	Ghana	West Africa	Korle-Bu Teaching Hospital, Southern Ghana	2020	NA	NA
Ducancelle, A.	Cameroon	Central Africa	Tokombere	2013	2009	2010
Ekouevi, D.K.	Togo	West Africa	Lome, Tsevie, Atakpame, Sokode, Kara and Dapaong Cities	2020	2017	2017
Etti, M.	Uganda	Eastern and Southern Africa	Kawempe National Referral Hospital	2021	NA	NA
Firde, M.	Ethiopia	Eastern and Southern Africa	Shone Hospital in Hadiya Zone, Southern Ethiopia	2022	2020	2020
Fomulu, N.J.	Cameroon	Central Africa	Yaounde University Teaching Hospital, Biyem-Assi (BADH) and the Cite Verte District Hospitals (CVDH) of Yaounde, the capital of Cameroon	2013	2011	2012
Fowotade, A.	Nigeria	West Africa	University College Hospital, Ibadan	2021	2018	2019
Gedefaw, G.	Ethiopia	Eastern and Southern Africa	Felegehiwot referral hospital	2019	2018	2018
Geffert, K.	United Republic of Tanzania	Eastern and Southern Africa	Bugando Medical Centre, Mwanza City	2020	2014	2015
Genetu, K.	Ethiopia	Eastern and Southern Africa	Tikur Anbessa Specialized Hospital, Zewuditu Memorial Hospital, Ghandi Memorial Hospital, and St. Petros Hospital; all in Addis Ababa	2022	2021	2021
Guingane, A.	Burkina Faso	West Africa	Baskuy, Ouagadougou	2020	2014	2016
Ifeorah, I. M.	Nigeria	West Africa	Ade-Oyo State Hospital and University College Hospital, Ibadan	2017	2012	2013

Itou-Ngaporo, A.	Republic of Congo	Central Africa	Four health centres in Brazaville: BISSITA, Jeanne Vialle, Marien Ngouabi and CHU	2016	2014	2014
Joseph Davey, D.	South Africa	Eastern and Southern Africa	Gugulethu Midwife Obstetrics Unit, Cape Town	2022	2020	2022
Kassa, D.	Ethiopia	Eastern and Southern Africa	12 major cities of Ethiopia	2019	2005	2014
Kirkbak, A.	South Sudan	Eastern and Southern Africa	Juba Teaching Hospital, Juba County, Central Equatoria State	2017	2012	2013
Kolawole, O.M.	Nigeria	West Africa	Ladoke Akintola University Teaching Hospital, Osogbo City, Osun state, South-western Nigeria	2012	2010	2011
Komas, N.P.	Central African Republic	Central Africa	All six public health structures with maternity wards in Bangui	2018	2010	2010
Loarec, A.	Mozambique	Eastern and Southern Africa	Chamanculo General Hospital, Maputo	2022	2017	2019
Loriette, M	Cameroon	Central Africa	Seven district health facilities in Tokombere district	2015	2009	2013
Lunel-Fabiani, F.	Cameroon	Central Africa	North Cameroon	2019	2009	2015
Manyahi, J.	United Republic of Tanzania	Eastern and Southern Africa	Temeke hospital<br>Zakheem Health Center<br>Kizuiani Health Center	2017	2014	2014
Marama, T.	Ethiopia	Eastern and Southern Africa	Jinka Hospital, Jinka Town (capital of the south Omo zone in southern Ethiopia)	2020	2020	2020
Meier-Stephenson, V.	Ethiopia	Eastern and Southern Africa	Gondar	2018	2016	2016
Meier-Stephenson, V	Ethiopia	Eastern and Southern Africa	University of Gondar Hospital, Gondar	2020	2016	2016
Metaferia, Y.	Ethiopia	Eastern and Southern Africa	Hawassa University referral hospital, Hawassa	2016	2015	2015
Mhata, P	Namibia	Eastern and Southern Africa	Erong, Khomas, Ohangwena, Omusati, Oshana, Oshikoto and Zambezi regions	2017	2013	2013
Mugabiirwe, N.	Uganda	Eastern and Southern Africa	Kyazanga HCIV Antenatal Clinic, Lwengo District	2022	2021	2021

Mustapha, G.U.	Nigeria	West Africa	Primary Health Centres (PHCs) of Gamawa LGA, Bauchi State	2020	2018	2018
Mutagoma, M.	Rwanda	Eastern and Southern Africa	Rwanda (National Study)	2017	2011	2011
Ngaira, J.A.	Kenya	Eastern and Southern Africa	Mbagathi District Hospital	2016	2014	2014
Nguyen, A.	Mozambique	Eastern and Southern Africa	Chamanculo General Hospital, Maputo City	2019	2017	2019
Nlinwe, NO.	Cameroon	Central Africa	Bamenda Regional Hospital, Mezam I Sub Division	2021	2020	2020
Oliveira, D.	Angola	Central Africa	Irene Neto Maternity of Lubango city	2020	2016	2017
Oluremi, AS.	Nigeria	West Africa	Adeoyo Maternity Teaching Hospital Ibadan, Oyo State	2020	2019	2019
Osazuwa, F	Nigeria	West Africa	ANC of Abaji General Hospital, Abaji	2012	2010	2011
Peliganga, L	Angola	Central Africa	Hospital Geral do Bie, Kuito, Luanda	2022	2007	2007
Rashid, S., C.	United Republic of Tanzania	Eastern and Southern Africa	Muhimbili National Hospital, Dar es Salaam	2014	2010	2010
Roble, A.K.	Ethiopia	Eastern and Southern Africa	Jigjiga University Sheik Hassen Bare Referral Hospital<br>Karamara Hospital<br>Jigjiga Health Centre	2020	2019	2019
Shimakawa, Y.	Cameroon	Central Africa	Tokombere District Hospital, Tokombere District	2022	2009	2016
Tamandjou, C., S	Namibia	Eastern and Southern Africa	Antenatal clinics in Windhoek, Namibia	2019	NA	NA
Tanjong, R.E.	Cameroon	Central Africa	Buea Health District Antenatal Clinics	2016	2010	2010
Tegegne, D	Ethiopia	Eastern and Southern Africa	St. Paul's Hospital Millenium Medical College and Selam Health Centre, Addis Ababa	2014	2012	2012
Thomson P	Democratic Republic of the Congo	Central Africa	Binza and Kingasani Maternity Centres, Kinshasa	2021	2018	2019
Thumbiran, N.	South Africa	Eastern and Southern Africa	KwaZulu-Natal Region	2014	2009	2009
Torimiro, J.N.	Cameroon	Central Africa	Yaounde, Cameroon	2018	2011	2015
Umare, A.	Ethiopia	Eastern and Southern Africa	Referral hospital, Deder, Eastern Ethiopia	2016	2015	2015

Utoo, B2	Nigeria	West Africa	Sacred Heart Hopsital, Obudu, South Nigeria	2013	2010	2010
Vueba, A. N.	Angola	Central Africa	Lucrecia Palm Maternity Hospital, Luanda	2021	2016	2017
Volker, F., P.	Ghana	West Africa	Pregnant women from the Jomoro, Nzema East Municipal, Ahanta West, and Ellembelle district (Western Region of Ghana)	2017	2011	2012
Wakjira, M.	Ethiopia	Eastern and Southern Africa	Ambo town	2022	2019	2019
Woldesonbet, Z	Ethiopia	Eastern and Southern Africa	Addis Ababa	2016	2014	2014
Woldesonbet, Z.D.	Ethiopia	Eastern and Southern Africa	Bishoftu Hospital	2016	2014	2014
Yohanes, T.	Ethiopia	Eastern and Southern Africa	Arba Minch Hospital, Arba Minch Town	2016	2015	2015
Zenebe, Y.	Ethiopia	Eastern and Southern Africa	Bahir Dar City referral hospital and three randomly selected health centres, Amhara National Regional State	2014	2013	2013

S11 Appendix. Forest plot of HBsAg prevalence in pregnant women by WHO Africa sub-region Surveys taken from the 2014-2023 dataset and results stratified by WHO sub-region; [A] Eastern and Southern Africa; [B] Central Africa and [C] West Africa. Pooled estimates are from a binomial mixed model.

A: Eastern and Southern Africa

Study	Country	Location	Mid-study year	HBsAg +ve/ tested	Prevalence (%)	95% CI
ESA16	Ethiopia	12 major cities	2009	497/4887	10.2	[9.3; 11.1]
ESA21	Ethiopia	Gambo	2011	13/574	2.3	[1.2; 3.8]
ESA22	Ethiopia	Addis Ababa	2012	8/265	3.0	[1.3; 5.9]
ESA25	Ethiopia	Bahir Dar	2013	12/318	3.8	[2.0; 6.5]
ESA28	Ethiopia	East Wollega Zone	2014	10/421	2.4	[1.1; 4.3]
ESA31	Ethiopia	Addis Ababa	2014	13/215	6.0	[3.3; 10.1]
ESA29	Ethiopia	Bishoftu	2014	11/202	5.4	[2.7; 9.5]
ESA37	Ethiopia	Tigray Province	2015	18/328	5.5	[3.3; 8.5]
ESA36	Ethiopia	Hawassa	2015	21/269	7.8	[4.9; 11.7]
ESA35	Ethiopia	Deder	2015	22/318	6.9	[4.4; 10.3]
ESA34	Ethiopia	Arba Minch	2015	10/232	4.3	[2.1; 7.8]
ESA38	Ethiopia	Gondar	2016	2/200	1.0	[0.1; 3.6]
ESA41	Ethiopia	Azer	2017	10/222	4.5	[2.2; 8.1]
ESA40	Ethiopia	Debre Markos	2017	28/338	8.3	[5.6; 11.8]
ESA45	Ethiopia	Wolaita Zone	2018	49/675	7.3	[5.4; 9.5]
ESA46	Ethiopia	Amhara State	2018	85/1846	4.6	[3.7; 5.7]
ESA42	Ethiopia	Bahir Dar	2018	16/338	4.7	[2.7; 7.6]
ESA47	Ethiopia	Jigjiga	2019	50/589	8.5	[6.4; 11.0]
ESA48	Ethiopia	Ambo	2019	18/361	5.0	[3.0; 7.8]
ESA49	Ethiopia	Addis Ababa	2020	369/12318	3.0	[2.7; 3.3]
ESA52	Ethiopia	Hadiya Zone	2020	12/245	4.9	[2.6; 8.4]
ESA50	Ethiopia	Jinka	2020	38/422	9.0	[6.5; 12.2]
ESA51	Ethiopia	West Hararghe Zone	2020	24/300	8.0	[5.2; 11.7]
ESA53	Ethiopia	Addis Ababa	2021	9/281	3.2	[1.5; 6.0]
ESA32	Kenya	Mbagathi District	2014	11/287	3.8	[1.9; 6.8]
ESA44	Mozambique	Maputo	2018	270/6775	4.0	[3.5; 4.5]
ESA26	Namibia	4 regions	2013	836/11390	7.3	[6.9; 7.8]
ESA39	Namibia	Windhoek	2016	28/515	5.4	[3.6; 7.8]
ESA20	Rwanda	All provinces	2011	486/13121	3.7	[3.4; 4.0]
ESA17	South Africa	KwaZulu Natal	2009	14/294	4.8	[2.6; 7.9]
ESA19	South Africa	Tshwane	2010	18/2368	0.8	[0.5; 1.2]
ESA30	South Africa	Cape Town	2014	6/134	4.5	[1.7; 9.5]
ESA54	South Africa	Cape Town	2021	8/1194	0.7	[0.3; 1.3]
ESA24	South Sudan	Juba	2013	31/280	11.1	[7.6; 15.3]
ESA18	Tanzania	Dar es Salaam	2010	12/310	3.9	[2.0; 6.7]
ESA33	Tanzania	Mwanza	2014	22/743	3.0	[1.9; 4.4]
ESA27	Tanzania	Dar es Salaam	2014	20/249	8.0	[5.0; 12.1]
ESA23	Uganda	Gulu	2012	47/397	11.8	[8.8; 15.4]
ESA43	Uganda	Kampala	2018	133/6062	2.2	[1.8; 2.6]
ESA55	Uganda	Lwengo District	2021	8/384	2.1	[0.9; 4.1]
ESA56	Uganda	Kasese District	2021	50/1065	4.7	[3.5; 6.1]
**Random effects model**					**4.4**	**[3.6; 5.4]**

Heterogeneity: $I^2 = 95.5\%$, $\tau^2 = 0.3759$, $p < 0.0001$

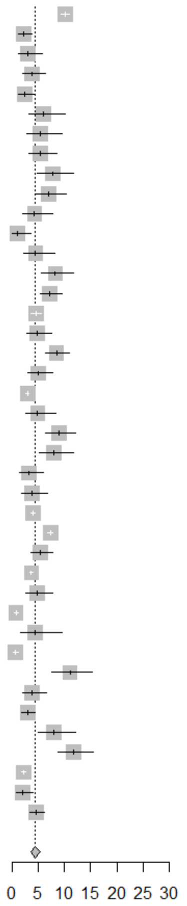

B: Central Africa

Study	Country	Location	Mid-study year	HBsAg +ve/ tested	Prevalence (%)	95% CI
CA05	Angola	Luanda	2007	88/1019	8.6	[7.0; 10.5]
CA16	Angola	Luanda	2016	226/878	25.7	[22.9; 28.8]
CA17	Angola	Lubango	2017	43/500	8.6	[6.3; 11.4]
CA07	CAR	Bangui	2010	71/874	8.1	[6.4; 10.1]
CA08	Cameroon	Buea	2010	49/406	12.1	[9.1; 15.6]
CA12	Cameroon	Tokombere	2012	3901/22243	17.5	[17.0; 18.0]
CA13	Cameroon	Yaounde	2013	64/1005	6.4	[4.9; 8.1]
CA15	Cameroon	Western Cameroon	2014	308/7069	4.4	[3.9; 4.9]
CA19	Cameroon	Mezam Division	2020	11/221	5.0	[2.5; 8.7]
CA20	Cameroon	Centre Region	2020	133/1992	6.7	[5.6; 7.9]
CA14	Congo	Brazzaville	2014	38/437	8.7	[6.2; 11.7]
CA18	DRC	Kinshasa	2018	109/4016	2.7	[2.2; 3.3]
CA22	DRC	Kinshasa	2021	64/3135	2.0	[1.6; 2.6]
CA21	Gabon	Franceville	2021	35/901	3.9	[2.7; 5.4]
**Random effects model**					**7.0**	**[4.9; 10.0]**

Heterogeneity: $I^2 = 99.2\%$, $\tau^2 = 0.5263$, $p = 0$

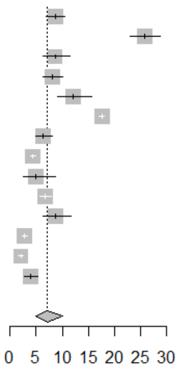

C: West Africa

Study	Country	Location	Mid-study year	HBsAg +ve/ tested	Prevalence (%)	95% CI
WA24	Benin	Atacora District	2011	44/283	15.5	[11.5; 20.3]
WA29	Burkina Faso	Ouagadogou	2015	75/1580	4.7	[3.8; 5.9]
WA38	Burkina Faso	Yako District	2021	106/1622	6.5	[5.4; 7.8]
WA28	Gambia	Banjul	2015	39/426	9.2	[6.6; 12.3]
WA37	Gambia	Multiple sites	2020	435/9681	4.5	[4.1; 4.9]
WA26	Ghana	Western Region	2011	29/174	16.7	[11.5; 23.1]
WA30	Ghana	Tamale	2016	356/8473	4.2	[3.8; 4.7]
WA31	Ghana	Accra	2017	17/221	7.7	[4.5; 12.0]
WA34	Ghana	South Tongu District	2018	11/250	4.4	[2.2; 7.7]
WA33	Guinea	Conakry	2018	36/305	11.8	[8.4; 16.0]
WA39	Mali	Bamako	2022	84/998	8.4	[6.8; 10.3]
WA25	Nigeria	Kaduna	2011	31/800	3.9	[2.6; 5.5]
WA23	Nigeria	Ibadan	2011	107/658	16.3	[13.5; 19.3]
WA27	Nigeria	Ibadan	2013	15/272	5.5	[3.1; 8.9]
WA35	Nigeria	Ibadan	2018	18/172	10.5	[6.3; 16.0]
WA32	Nigeria	Bauchi State	2018	14/210	6.7	[3.7; 10.9]
WA36	Nigeria	Oyo State	2019	57/904	6.3	[4.8; 8.1]
**Random effects model**					**7.5**	**[5.9; 9.4]**

Heterogeneity: $I^2 = 95.1\%$, $\tau^2 = 0.2478$, $p < 0.0001$

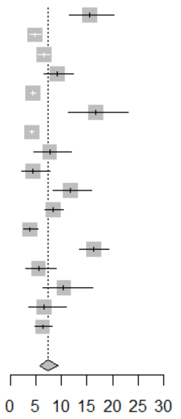

S12 Appendix. Plot of HBsAg prevalence stratified by quality score quartile

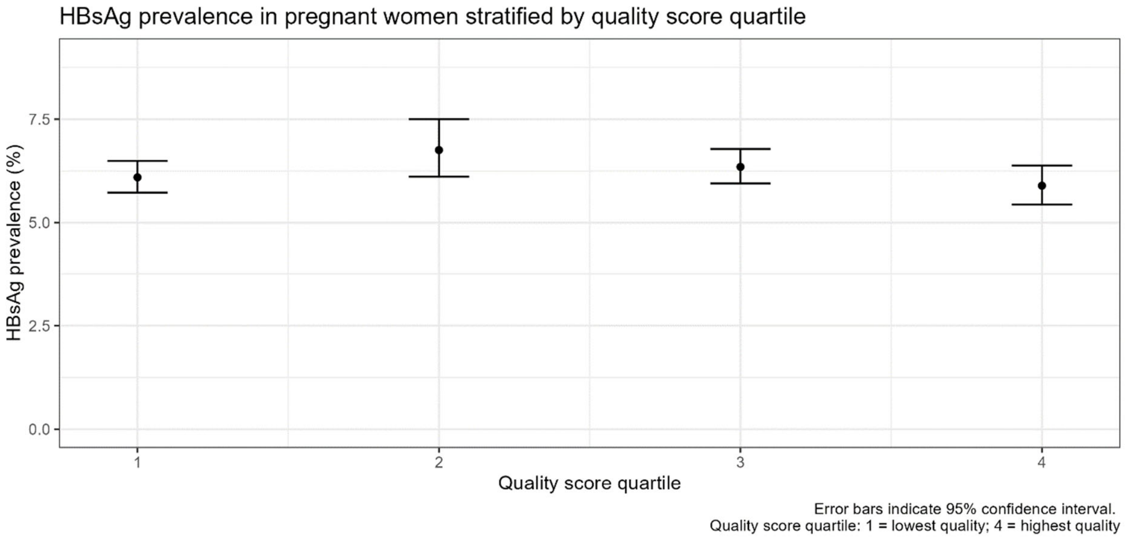

S13 Appendix. Meta-regression of characteristics associated with HBsAg prevalence in pregnant women attending for antenatal care

	Univariate		Multivariable	
**Characteristic**	**Odds ratio (95% CI)**	**p-value**	**Odds ratio (95% CI)**	**p-value**
**WHO region**				
Eastern and Southern Africa	Reference	-	Reference	-
Central Africa	1·78 (1·27-2·49)	<0·001	1·76 (1·27-2·45)	<0·001
West Africa	1·74 (1·33-2·29)	<0·001	1·61 (1·23-2·10)	<0·001
**Test type**				
ELISA/EIA - 78 studies	Reference	-	Reference	-
CLIA - 5 studies	0·46 (0·24-0·88)	0·020	0·68 (0·37-1·25)	0·20
RDT - 30 studies	0·76 (0·57-1·02)	0·071	0·85 (0·63-1·13)	0·30
**Interval from vaccine introduction to study start date (per 1 year increase)**	0·98 (0·96-0·99)	<0·001	0·98 (0·97-1·00)	0·010

S14 Appendix. Forest plot of HBeAg prevalence in pregnant women, stratified by WHO Africa sub-region

A: Eastern and Southern Africa

Study	Country	Location	Mid-study year	HBeAg +ve/ tested	Prevalence (%)	95% CI
ESA44	Mozambique	Maputo	2018	24/265	9.1	[5.9; 13.2]
ESA20	Rwanda	All provinces	2011	40/178	22.5	[16.6; 29.3]
ESA17	South Africa	KwaZulu Natal	2009	0/14	0.0	[0.0; 23.2]
ESA30	South Africa	Cape Town	2014	1/6	16.7	[0.4; 64.1]
ESA18	Tanzania	Dar es Salaam	2010	0/12	0.0	[0.0; 26.5]
ESA33	Tanzania	Mwanza	2014	2/22	9.1	[1.1; 29.2]
ESA23	Uganda	Gulu	2012	7/47	14.9	[6.2; 28.3]
**Random effects model**					**11.4**	**[6.6; 19.1]**

Heterogeneity: $I^2 = 60.8\%$, $\tau^2 = 0.2408$, $p = 0.0179$

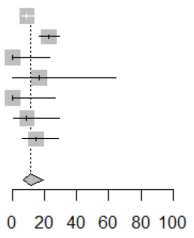

B: Central Africa

Study	Country	Location	Mid-study year	HBeAg +ve/ tested	Prevalence (%)	95% CI
CA05	Angola	Luanda	2007	5/68	7.4	[2.4; 16.3]
CA16	Angola	Luanda	2016	78/226	34.5	[28.3; 41.1]
CA12	Cameroon	Tokombere	2012	128/597	21.4	[18.2; 25.0]
CA20	Cameroon	Centre Region	2020	12/133	9.0	[4.7; 15.2]
CA18	DRC	Kinshasa	2018	9/90	10.0	[4.7; 18.1]
CA21	Gabon	Franceville	2021	1/35	2.9	[0.1; 14.9]
**Random effects model**					**12.5**	**[6.6; 22.5]**

Heterogeneity: $I^2 = 90.3\%$, $\tau^2 = 0.6187$, $p < 0.0001$

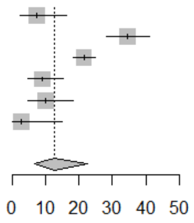

C: West Africa

Study	Country	Location	Mid-study year	HBeAg +ve/ tested	Prevalence (%)	95% CI
WA24	Benin	Atacora District	2011	5/44	11.4	[3.8; 24.6]
WA38	Burkina Faso	Yako District	2021	23/102	22.5	[14.9; 31.9]
WA26	Ghana	Western Region	2011	5/23	21.7	[7.5; 43.7]
WA27	Nigeria	Ibadan	2013	1/15	6.7	[0.2; 31.9]
**Random effects model**					**18.1**	**[11.5; 27.2]**

Heterogeneity: $I^2 = 22.3\%$, $\tau^2 = 0.0161$, $p = 0.2769$

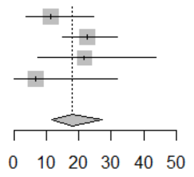

S15 Appendix: Characteristics of included samples assessing mother to child transmission, stratified by vaccine status

	Vaccine status assessed				
**Characteristic**	**Birth dose (Hep-BD) within 24 hours**	**Delayed birth dose (>24 hours and <1 week)**	**Hep-B3 (without birth dose)**	**No HBV vaccine**	**Overall**
**Number of samples**	7	4	5	6	22
**Countries included**	Cameroon , DRC, Ethiopia, Gambia, Ivory Coast, Mozambique, South Africa	Cameroon, DRC, Gambia	DRC, Ethiopia, Gambia, Ivory Coast, Mozambique	Ethiopia, Gambia, Mozambique, Senegal, Tanzania	
**Total number of pregnant women tested for HBsAg**	558	62	250	410	1280
**Total number of infants testing positive for HBsAg**	16 (2·9%)	4 (6.5%)	20 (8·0%)	109 (26·6%)	149 (11·6%)
**Number of mother-infant pairs tested per study** (median; IQR)	74 (41-121)	13 (7-22)	35(4-81)	39 (23-53)	37 (11-79)
**Infant age (months) when tested** (median; IQR)	8·0 (6·3-9·0)	6.0 (6.0-7.5)	9·0 (6·0-9·0)	7·0 (6·0-8·8)	8·0 (6·0-9·0)

S16 Appendix. Details of included MTCT studies

Title	Country	Vaccine subgroup	start_dt (mm/yyy)	stop_dt (mm/yyyy)	Age at infant testing (months)	HBsAg test type (infant)	HBsAg test type (mother)	HBsAg +ve infants	No. infants tested	HIV prevalence (mothers) n/N	HBeAg prevalence (mothers)	Provision of maternal antivirals
**Arefaine, M. (2023)**	Ethiopia	Birth dose	01/2019	05/2021	9	ELISA/EIA	Not reported	5	139	Not reported	Not reported	Not reported
**Arefaine, M. (2023)**	Ethiopia	HepB3 (not BD)	01/2019	05/2021	9	ELISA/EIA	Not reported	9	35	Not reported	Not reported	Not reported
**Bayu, H. (2020)**	Ethiopia	No vaccine	01/2018	09/2019	9	ELISA/EIA	Not reported	95	249	55/401 in underlying sample	Not reported	54/271
**Chotun, N. (2017)**	South Africa	Birth dose	06/2014	11/2014	7	RDT	RDT	0	4	0/4	1/4	2/4
**Ekra, D. (2008)**	Ivory Coast	Birth dose	01/2001	09/2002	9	ELISA/EIA	ELISA/EIA	9	156	Not reported	24/156	0/156
**Ekra, D. (2008)**	Ivory Coast	HepB3 (not BD)	01/2001	09/2002	9	ELISA/EIA	ELISA/EIA	10	129	Not reported	17/129	0/129
**Kibassa, C. (2004)**	Tanzania	No vaccine	01/2001	09/2002	6	ELISA/EIA	ELISA/EIA	6	51	Not reported	Not reported	Not reported
**Loarec, A. (2022)**	Mozambique	Birth dose	11/2017	09/2019	9	Not reported	RDT	0	103	98/270 in underlying sample	24/265 in underlying sample	36/103
**Loarec, A. (2022)**	Mozambique	Delayed birth dose	11/2017	09/2019	9	Not reported	RDT	1	3	98/270 in underlying sample	24/265 in underlying sample	1/3

**Loarec, A. (2022)**	Mozambique	HepB3 (not BD)	11/2017	09/2019	9	Not reported	Not reported	0	1	98/270 in underlying sample	24/265 in underlying sample	102/270 in underlying sample
**Loarec, A. (2022)**	Mozambique	No vaccine	11/2017	09/2019	9	Not reported	Not reported	0	27	98/270 in underlying sample	24/265 in underlying sample	10/27
**Menendez, C. (1999)**	Tanzania	No vaccine	01/1995	10/1995	8	ELISA/EIA	ELISA/EIA	4	53	Not reported	12/62	Not reported
**Ndow, G. (2023)**	Gambia	Birth dose	2020	2022	6	Not reported	RDT	2	74	Not reported	2/60	Not reported
**Ndow, G. (2023)**	Gambia	Delayed birth dose	2020	2022	6	Not reported	RDT	2	33	Not reported	7/103	Not reported
**Ndow, G. (2023)**	Gambia	HepB3 (not BD)	2020	2022	6	Not reported	RDT	1	81	Not reported	7/103	Not reported
**Ndow, G. Senegal**	Gambia	No vaccine	2020	2022	6	Not reported	RDT	1	9	Not reported	Not reported	Not reported
**Roingeard (1993)**	Senegal	No vaccine	NR	NR	6	ELISA/EIA	ELISA/EIA	3	21	Not reported	Not reported	Not reported
**Shimakawa, Y. (2022) Cameroon**	Cameroon	Delayed birth dose	01/2009	12/2016	7-12m	ELISA/EIA	ELISA/EIA	1	18	1/605 in underlying sample	128/597 in underlying sample	Not reported
**Shimakawa, Y. (2022) Cameroon**	Cameroon	Birth dose	01/2009	12/2016	7-12m	ELISA/EIA	ELISA/EIA	0	43	1/605 in underlying sample	128/597 underlying sample	Not reported

**Thompson, P. (2021) Democratic Republic of Congo**	DRC	Birth dose	09/2018	02/2019	6	RDT	RDT	0	39	1/90 in underlying sample	10/90 in underlying sample	7/53 in underlying sample
**Thompson, P. (2021) Democratic Republic of Congo**	DRC	Delayed birth dose	09/2018	02/2019	6	RDT	RDT	0	8	1/90 in underlying sample	10/90 in underlying sample	7/53 in underlying sample
**Thompson, P. (2021) Democratic Republic of Congo**	DRC	HepB3 (not BD)	09/2018	02/2019	6	RDT	RDT	0	4	1/90 in underlying sample	10/90 in underlying sample	7/53 in underlying sample

S17 Appendix. Forest plot of mother to child transmission rate, stratified by intervention sub-groups: [A] Birth dose; [B] Delayed birth dose; [C] HepB3 without a birth dose; [D] No vaccination; [E] Received maternal antiviral prophylaxis

[A] Birth dose (HEP-BD)

Study	Publication year	Country	+ve infants / total tested	Transmission (95% CI)
ESA30	2017	South Africa	0/4	0.0 (0.0-60.2)
CA12	2022	Cameroon	0/43	0.0 (0.0-8.2)
ESA44	2022	Mozambique	0/103	0.0 (0.0-3.5)
CA18	2021	DRC	0/39	0.0 (0.0-9.0)
WA37	2023	Senegal	2/74	2.7 (0.3-9.4)
ESA49	2023	Ethiopia	5/139	3.6 (1.2-8.2)
WA09	2008	Ivory Coast	9/156	5.8 (2.7-10.7)
**Random effects model**				**1.8 (0.5-6.1)**

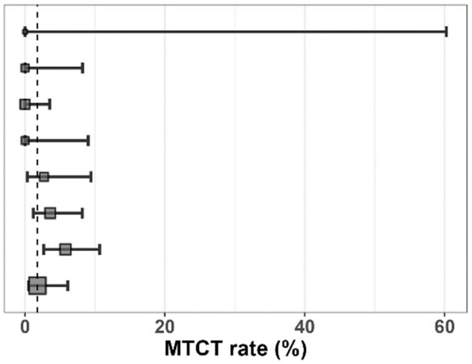

[B] Delayed birth dose

Study	Publication year	Country	+ve infants / total tested	Transmission (95% CI)
CA18	2021	DRC	0/8	0.0 (0.0-36.9)
CA12	2022	Cameroon	1/18	5.6 (0.1-27.3)
WA37	2023	Senegal	2/33	6.1 (0.7-20.2)
ESA44	2022	Mozambique	1/3	33.3 (0.8-90.6
**Random effects model**				**6.4 (2.4-16.0)**

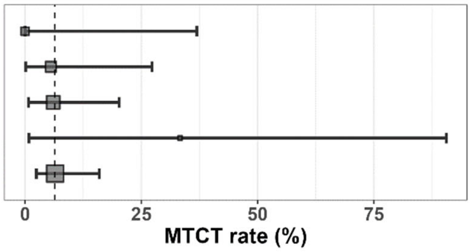

[C] HEPB3 (not birth dose)

Study	Publication year	Country	+ve infants / total tested	Transmission (95% CI)
CA18	2021	DRC	0/4	0.0 (0.0-60.2)
ESA44	2022	Mozambique	0/1	0.0 (0.0-97.5)
WA37	2023	Senegal	1/81	1.2 (0.0-6.7)
WA09	2008	Ivory Coast	10/129	7.8 (3.8-13.8)
ESA49	2023	Ethiopia	9/35	25.7 (12.5-43.3)
**Random effects model**				**6.2 (1.5-21.8)**

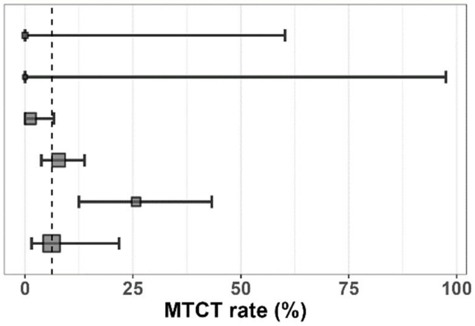

[D] No HBV vaccination

Study	Publication year	Country	+ve infants / total tested	Transmission (95% CI)
ESA08	1999	Tanzania	4/53	7.5 (2.1-18.2)
WA37	2023	Senegal	1/9	11.1 (0.3-48.2)
ESA09	2004	Tanzania	6/51	11.8 (4.4-23.9)
WA40	1993	Senegal	3/21	14.3 (3.0-36.3)
ESA57	2020	Ethiopia	95/249	38.1 (32.1-44.5)
**Random effects model**				**11.2 (4.6-25.1)**

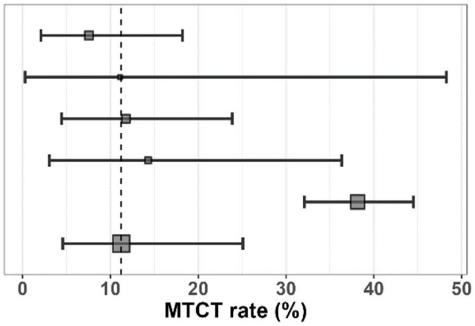

[E] Maternal antiviral therapy

Study	Publication year	Country	+ve infants / total tested	Transmission (95% CI)
CA18	2021	DR Congo	0/7	0.0 (0.0-41.0)
ESA30	2017	South Africa	0/2	0.0 (0.0-84.2)
ESA44	2022	Mozambique	1/47	2.1 (0.1-11.3)
**Random effects model**				**1.8 (0.3-11.6)**

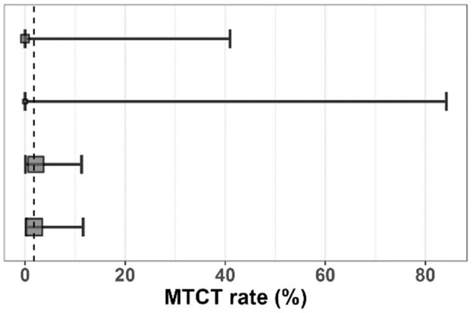

S18 Appendix. Mother to child transmission stratified by vaccination subgroups, excluding cohorts which offered maternal antiviral prophylaxis: [A] Birth dose; [B] Delayed birth dose; [C] HepB3 without a birth dose; [D] No vaccination

[A] Birth dose (HEP-BD), excluding cohorts with maternal antiviral prophylaxis

Study	Publication year	Country	+ve infants / total tested	Transmission (95% CI)
CA12	2022	Cameroon	0/43	0.0 (0.0-8.2)
WA37	2023	Senegal	2/74	2.7 (0.3-9.4)
ESA49	2023	Ethiopia	5/139	3.6 (1.2-8.2)
WA09	2008	Ivory Coast	9/156	5.8 (2.7-10.7)
**Random effects model**				**3.9 (2.4-6.2)**

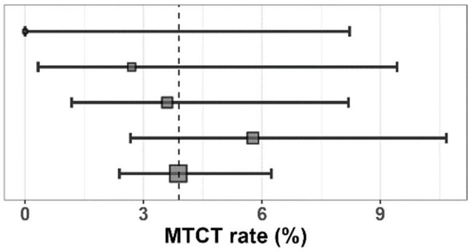

[B] Delayed birth dose, excluding cohorts with maternal antiviral prophylaxis

Study	Publication year	Country	+ve infants / total tested	Transmission (95% CI)
CA12	2022	Cameroon	1/18	5.6 (0.1-27.3)
WA37	2023	Senegal	2/33	6.1 (0.7-20.2)
**Random effects model**				**5.9 (1.9-16.7)**

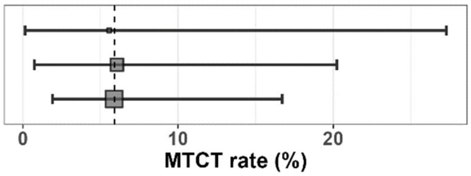

[C] HEPB3 (not birth dose), excluding cohorts with maternal antiviral therapy

Study	Publication year	Country	+ve infants / total tested	Transmission (95% CI)
WA37	2023	Senegal	1/81	1.2 (0.0-6.7)
WA09	2008	Ivory Coast	10/129	7.8 (3.8-13.8)
ESA49	2023	Ethiopia	9/35	25.7 (12.5-43.3)
**Random effects model**				**7.3 (1.8-25.4)**

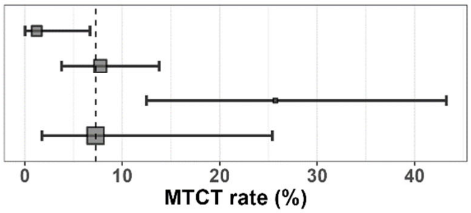

[D] No HBV vaccination, excluding cohorts with maternal antiviral therapy

Study	Publication year	Country	+ve infants / total tested	Transmission (95% CI)
ESA08	1999	Tanzania	4/53	7.5 (2.1-18.2)
WA37	2023	Senegal	1/9	11.1 (0.3-48.2)
ESA09	2004	Tanzania	6/51	11.8 (4.4-23.9)
WA40	1993	Senegal	3/21	14.3 (3.0-36.3)
ESA57	2020	Ethiopia	95/249	38.1 (32.1-44.5)
**Random effects model**				**15.5 (7.5-29.4)**

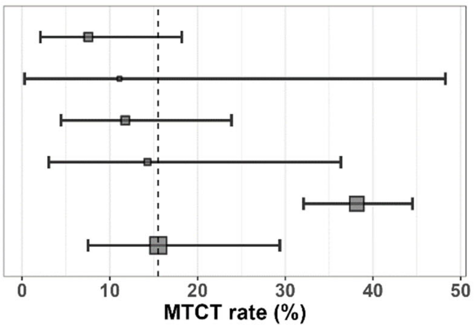

Supplement: Supplementary appendix [file mmc1.pdf]
